# Identification of FadAB Complexes Involved in Fatty Acid β-Oxidation in *Streptomyces coelicolor* and Construction of a Triacylglycerol Overproducing strain

**DOI:** 10.3389/fmicb.2017.01428

**Published:** 2017-08-02

**Authors:** Simón Menendez-Bravo, Julián Paganini, Claudio Avignone-Rossa, Hugo Gramajo, Ana Arabolaza

**Affiliations:** ^1^Microbiology Division, Instituto de Biología Molecular y Celular de Rosario, Consejo Nacional de Investigaciones Científicas y Técnicas, Facultad de Ciencias Bioquímicas y Farmacéuticas, Universidad Nacional de Rosario Rosario, Argentina; ^2^Department of Microbial Sciences, School of Biosciences and Medicine, University of Surrey Guildford, United Kingdom

**Keywords:** *Streptomyces coelicolor*, triacylglycerol accumulation, beta-oxidation of fatty acids, neutral lipid, chemostat system

## Abstract

Oleaginous microorganisms represent possible platforms for the sustainable production of oleochemicals and biofuels due to their metabolic robustness and the possibility to be engineered. *Streptomyces coelicolor* is among the narrow group of prokaryotes capable of accumulating triacylglycerol (TAG) as carbon and energy reserve. Although the pathways for TAG biosynthesis in this organism have been widely addressed, the set of genes required for their breakdown have remained elusive so far. Here, we identified and characterized three gene clusters involved in the β-oxidation of fatty acids (FA). The role of each of the three different *S. coelicolor* FadAB proteins in FA catabolism was confirmed by complementation of an *Escherichia coli*Δ*fadBA* mutant strain deficient in β-oxidation. In *S. coelicolor*, the expression profile of the three gene clusters showed variation related with the stage of growth and the presence of FA in media. Flux balance analyses using a corrected version of the current *S. coelicolor* metabolic model containing detailed TAG biosynthesis reactions suggested the relevance of the identified *fadAB* genes in the accumulation of TAG. Thus, through the construction and analysis of *fadAB* knockout mutant strains, we obtained an *S. coelicolor* mutant that showed a 4.3-fold increase in the TAG content compared to the wild type strain grown under the same culture conditions.

## Introduction

Species of the genus *Streptomyces* produce the vast majority of clinically used antibiotics and also belong to the narrow group of prokaryotes capable of accumulating triacylglycerol (TAG) (Olukoshi and Packter, 1994; Gago et al., 2011; Alvarez, 2016), which is a metabolic feature typically found in eukaryotes. TAG storage is of utmost interest since bio-oil based chemistry is considered one of the most promising alternatives to petroleum derivatives for the production of fuels and chemicals (Ledesma-Amaro and Nicaud, 2016). In recent years, research has focused on oleaginous microbes as they can be metabolically engineered to over-accumulate lipids, constituting a possible platform for sustainable bio-oleochemicals production (Peralta-Yahya et al., 2012; Lennen and Pfleger, 2013; Alvarez, 2016; Lynch, 2016).

Lipid metabolism in *Streptomyces coelicolor*, the model organism of the genus *Streptomyces*, has been partially characterized (Banchio and Gramajo, 1997; Hobbs et al., 1997; Arabolaza et al., 2008, 2010; Comba et al., 2013). This species can efficiently utilize exogenous fatty acids (FA), from C4 to C18, as sole carbon and energy source, combining simple diffusion and active transport mechanisms for the import of short- and long-chain FA, respectively (Banchio and Gramajo, 1997). When present in the medium, glucose stimulates the degradation of long-chain FA partly by increasing the uptake rate of FA and the activity of acyl-CoA synthetase (Banchio and Gramajo, 1997). As mentioned, this microorganism utilizes FA not only to synthesize membrane phospholipids but also to be incorporated into neutral lipid storage compounds, such as TAG (Olukoshi and Packter, 1994; Alvarez, 2016). Due to their extreme hydrophobicity, TAG are excellent reserve materials as they can be accumulated in large amounts without affecting cell osmolarity (Alvarez, 2016). In submerged liquid culture, TAG synthesis and cytoplasmic accumulation as lipid droplets usually occur during the post-exponential phase of growth (Olukoshi and Packter, 1994). In *S. coelicolor*, the diacylglycerol acyltransferase (DGAT) SCO0958, which uses acyl-CoAs as acyl donors and diacylglycerol (DAG) as acceptor, has been proposed to play a fundamental role in the final step of TAG biosynthesis (Arabolaza et al., 2008). In addition, other pathways in this bacterium have been suggested to substantially contribute to TAG biosynthesis (Arabolaza et al., 2008).

However, despite the focus on the identification of genes and enzymes involved in building up intracellular TAG, the breakdown of this neutral lipid is poorly understood. It is well-known that oleaginous bacteria are able to mobilize TAG under certain physiological or environmental conditions: during the late stationary phase of growth, in the absence of a carbon source, or under water stress conditions (Olukoshi and Packter, 1994; Alvarez, 2016). Since acyl moieties are highly reduced compounds, TAG mobilization is associated to high energy yields (Alvarez and Steinbüchel, 2002). However, regulatory signals and genes involved in the catabolism of TAG and FA in these bacteria have not been described so far.

In general, degradation of neutral lipids requires a variety of hydrolases and lipases to release free FA. Coenzyme-A (CoA) activated derivatives of FA are then broken down into acetyl-CoA by the β-oxidation pathway, which consists of five core catalytic functions that are highly conserved in all living organisms (Schulz, 1991; Yang and He, 1999). This pathway was first characterized in *Escherichia coli* (Yang et al., 1988, 1990; DiRusso, 1990; Black et al., 1992; Raman et al., 1997). The first committed step is the activation of free FA to their corresponding fatty acyl-CoA by the acyl-CoA synthetase FadD. After that, a cycle of four reactions begins with the generation of an unsaturation at the α-β position by the acyl-CoA dehydrogenase FadE (Ghisla and Thorpe, 2004). Then, the enoyl-CoA hydratase (ECH) hydroxylates the carbon chain at the β position and the hydroxyl group is subsequently oxidized by 3-hydroxyacyl-CoA dehydrogenase (3HCDH) to yield 3-ketoacyl-CoA. The ECH and the 3HCDH activities are catalyzed by the multifunctional protein FadB (Yang et al., 1988). Finally, the ketothiolase FadA cleaves the 3-ketoacyl-CoA between C2 and C3, releasing acetyl-CoA and a fatty acyl-CoA two carbons shorter than the initial molecule. In the oxidation of FA with even number of carbons, this cycle repeats until the whole molecule has been converted into acetyl-CoA. In *E. coli*, FadB and FadA form a heterotetrameric complex consisting of two copies of each enzyme (Pramanik et al., 1979; Kim and Battaile, 2002; Campbell et al., 2003). It is generally assumed that the basic metabolic pathway of FA catabolism in oleaginous bacteria is analogous to those reported in other better-studied microorganisms. However, an *in silico* search over *S. coelicolor* genome, one of the largest known prokaryotic genomes, reveals many potential β-oxidation gene homologs. To the best of our knowledge, it is not yet known which of these putative candidates actually play a role in FA β-oxidation.

In this work, we identified three gene clusters coding for FadAB complexes in *S. coelicolor*, determined the *in vivo* activity of the encoded proteins and analyzed the corresponding expression patterns in their natural host. To predict TAG over-accumulating phenotypes, we performed flux balance analysis (FBA) based on a corrected version of the current *S. coelicolor* metabolic model. Finally, we determined the role of the identified genes in TAG mobilization through the construction and analysis of a set of different *S. coelicolor* knockout mutant strains.

## Materials and methods

### Strains, media, and growth conditions

The strains and plasmids used in this study are described in Table 1. *E. coli* strains were grown either on solid or in liquid Luria–Bertani or M9 media at 37°C and supplemented when needed with the following antibiotics: 100 μg/ml ampicillin (Ap), 100 μg/ml apramycin (Am), 20 μg/ml chloramphenicol (Cm), or 50 μg/ml kanamycin (Kn). *Streptomyces* strains were grown at 30°C in rich liquid medium R5, or minimal SMM medium supplemented with glucose 0.1% (w/v) and casamino acids 0.2% (w/v); and on the rich solid medium MS (Kieser et al., 2000). For genomic DNA extraction, cells were grown on TSB medium. The antibiotics Am, hygromycin (Hyg), Kn, and spectinomycin (Spec) were added at final concentrations of 50, 100, 50, and 200 μg/ml, respectively, to solid medium.

**Table 1:** Strains and plasmids used in this study.

**Strain**	**Description**	**References**
***E. coli***		
DH5α	K-12 F^−^*lacU169 (φ80lacZΔM15) endA1 recA1 hsdR17 deoR supE44 thi-1-l2 gyrA96 relA1*	Hanahan, 1983
BL21	*F^−^ ompT gal dcm lon hsdS_*B*_(${\text{r}}_{B}^{-}$${\text{m}}_{B}^{-}$) λ(DE3)*	Novagen®
Δ*fadBA*k	BL21 *ΔfadBA::*Km^R^	This work
Δ*fadBA*	BL21 *ΔfadBA*	This work
ET 12567	*supE44 hsdS20 ara-14 proA2 lacY galK2 rpsL20 xyl-5 mtl-1 dam dcm hdsM;* Cm^R^	Kieser et al., 2000
***S. coelicolor***		
M145	Parental strain, SCP1^−^ SCP2^−^	Kieser et al., 2000
SMB01	M145 *attB_Φ*C*31_*::pSM14; Km^R^	This work
SMB02	M145 *attB_Φ*C*31_*::pSM15; Km^R^	This work
SMB03	M145 *attB_Φ*C*31_*::pSM16; Km^R^	This work
SMB04	M145 *attB_Φ*C*31_*::pSM17; Km^R^	This work
SMB05	M145 *attB_Φ*C*31_*::pSM18; Km^R^	This work
SMB06	M145 *attB_Φ*C*31_*::pSM19; Km^R^	This work
SMB07	M145 SCO6731::*Tn5062*; Am^R^	This work
SMB08	M145 SCO6788::*Tn5062*; Am^R^	This work
SMB09	M145 SCO6027-SCO6026::Km^R^	This work
SMB10	SMB09, SCO6731::*Tn5062*; Am^R^	This work
SMB11	SMB09, SCO6788::*Tn5062*; Am^R^	This work
SMB12	SMB07, SCO6788::*Tn5062*; Hyg^R^	This work
**PLASMID**		
pUZ8002	Helper plasmid, RK2 derivative with defective *oriT*, Km^R^	Kieser et al., 2000
pKOS111-47	Helper plasmid, RK2 derivative with defective *oriT*, Ap^R^	B. Julien (pers. commun.)
pSET151	*S. coelicolor* non-replicative vector	Bierman et al., 1992
pIJ699	Plasmid used as template for amplifying Spectinomycin cassette; Ap^R^ Spec^R^	Lyutzkanova et al., 1997
pSM01	pSET151 derivative vector containing a Spec resistance cassette flanked by Ups6027 and Dns6026 sequences, Spec^R^ Thio^R.^	This work
pQM5066	Plasmid carrying a copy of *Tn5066*; Hyg^R^	P. Dyson (pers. commun.)
pET28a	Phagemid vector for expression of recombinant proteins under control of strong T7 transcription signals.	Novagen®
pBluescript II SK	Phagemid vector; ApR	Agilent®
pSM02	pET28a derivative vector containing SCO6731 gene; Km^R^	This work
pSM03	pET28a derivative vector containing SCO6732 gene; Km^R^	This work
pSM04	pET28a derivative vector containing SCO6788 gene; Km^R^	This work
pSM05	pET28a derivative vector containing SCO6789 gene; Km^R^	This work
pSM06	pET28a derivative vector containing SCO6027 gene; Km^R^	This work
**PLASMID**		
pSM07	pET28a derivative vector containing SCO6026 gene; Km^R^	This work
pSM08	pET28a derivative vector containing SCO6731-SCO6732 operon; Km^R^	This work
pSM09	pET28a derivative vector containing SCO6788-SCO6789 operon; Km^R^	This work
pSM10	pET28a derivative vector containing SCO6027-SCO6026 operon; Km^R^	This work
pSM11	pBluescript derivative vector containing SCO6731-SCO6732 operon; Km^R^	This work
pSM12	pBluescript derivative vector containing SCO6788-SCO6789 operon; Km^R^	This work
pSM13	pBluescript derivative vector containing SCO6027-SCO6026 operon; Km^R^	This work
pCA75	*Streptomyces* integrative and conjugative vector based on φC31 phage integrase, contains *xylTE* genes; Km^R^	This work
pSM14	pCA75 derivative vector containing SCO6730 promoter with *xylTE* genes under its control.	This work
pSM15	pCA75 derivative vector containing SCO6788 promoter with *xylTE* genes under its control.	This work
pSM16	pCA75 derivative vector containing SCO6027 promoter with *xylTE* genes under its control.	This work
pCA73	*Streptomyces* integrative and conjugative vector based on φC31 phage integrase, contains *cbg* genes; Km^R^	This work
pSM17	pCA73 derivative vector containing SCO6730 promoter with the *cbg* gene under its control.	This work
pSM18	pCA73 derivative vector containing SCO6788 promoter with the *cbg* gene under its control.	This work
pSM19	pCA73 derivative vector containing SCO6027 promoter with the *cbg* gene under its control.	This work
pKD13	Template plasmid for amplification of the FRT-flanked kanamycin cassette; ApR KmR	Datsenko and Wanner, 2000
pKD46	Temperature-sensitive replication plasmid for Red recombinase expression; ApR	Datsenko and Wanner, 2000
pCP20	Temperature-sensitive replication plasmid for thermal induction of FLP synthesis; CmR ApR	Cherepanov and Wackernagel, 1995
pCR®-BluntII-TOPO	Vector used for cloning of blunt PCR products; KmR	Invitrogen®

### Strains construction

*E. coli*Δ*fadBA* strain was constructed by lambda-red mediated recombination as described previously (Datsenko and Wanner, 2000). fadBA_F and fadBA_R oligonucleotides (Table 2) were used to amplify the Kn resistance cassette of pKD13 plasmid. The Kn resistance cassette was deleted by FLP recombinase-mediated excision.

**Table 2:** Oligonucleotides used in this study.

**Name**	**Sequence**
SCO6731_F	AAAGGCCTGCATATGAGCACCGAAGC
SCO6731_R	ACTAGTTCAGATGCGCTCGACG
SCO6732_F	CACACCCATATGAGCACCGAGC
SCO6732_R	ACTAGTAAGGAGGCCGGTGAATGAT
SCO6788_F	CCCCATATGAGCACCGAAGCGT
SCO6788_R	GTACTAGTCGGCGGCAGCACG
SCO6789_F	TCACATATGGACACCACCGCACG
SCO6789_R	GGTACTAGTCGCCGGAGTCAGAAG
SCO6027_F	CATATGCCTCGTACCGTCAGGG
SCO6027_R	ACTAGTGCTCACTTGTCCCCCTC
SCO6026_F	GGACATATGAGCACCACCGCAGAGC
SCO6026_R	ACTAGTTCACGCCGGTACCGACGCC
P_SCO6730_F	GAATTCCACCCGTCGGCAACCCCTG
P_SCO6730_R	GGATCCGCAGCCCCCAGCTCTGTGA
P_SCO6785_F	TTAGGAATTCATAAGCGATCGCGGGAGAG
P_SCO6785_R	GCGGTCTAGAATGGGTTCCTCTCTCGG
P_SCO6027_F	GAATTCCGGATTCCGGCCAAGGTCG
P_SCO6027_R	GGATCCACGATGGCTCTCCTCCAGG
Ups_6026_F	AAGCTTAGGTCCTGGGCCGCCACGC
Ups_6026_R	GGATCCGAGCGGGTGAACGGGAAGC
Dns_6027_F	GGATCCTCAGCGCCGCCACCAGTAC
Dns_6027_R	GAATTCATCGTTCCGGGCAGGGGGTC
Spec_F	CGCCACCCCCGCCTCCAGGAACCGC
Spec_R	CGGAGCCCGGCCCTGGCAGGTGGAG
fadBA_F	AAGGCGTTCACGCCGCATCCGGCAAGTGGTTAAACCCGCTCAAACACCGTTGTAGGCTGGAGCTGCTTCG
fadBA_R	GACTCATCTGGTACGACCAGATCACCTTGCGGATTCAGGAGACTGACATGATTCCGGGGATCCGTCGACC
fadBA_check_F	AGATCTATGACGTATCTGGCAAACC
fadBA_check_R	CGCATCTTAGCACATCGTTCATCC
EZR1	ATGCGCTCCATCAAGAAGAG
EZL2	TCCAGCTCGACCAGGATG

*The restriction sites used for cloning are underlined*.

*S. coelicolor SCOfadAB_2*^−^,*SCOfadAB_3*^−^,*SCOfadAB_1*^−^*SCOfadAB_2*^−^,*SCOfadAB_2*^−^*SCOfadAB_3*^−^, and *SCOfadAB_1*^−^*SCOfadAB_3*^−^ knockout mutants were constructed using cosmids from the transposon mutant ordered cosmid library from *S. coelicolor* (Bishop et al., 2004; Herron et al., 2004; Fernández-Martínez et al., 2011). Cosmids 5F2A.1.G09 and 8A04.1.h08, carrying individual *Tn5062* insertions in SCO6731 and SCO6788, respectively, were introduced into *S. coelicolor* M145 by conjugation using *E. coli* ET12567/pKOS111-47; yielding the strains SMB07 and SMB08. Three independent Am^R^ Km^S^ exconjugants were isolated for each event and checked by PCR, verifying that the allelic replacement had occurred. The SCO6731 gene disruption was analyzed with the following primer pairs: SCO6731_F and EZL2 (Herron et al., 2004), which hybridizes in the 5′ end region of SCO6731 and in *Tn5062*, respectively, and EZR1 and SCO6731_R, which hybridizes in Tn5062 and in the 3′ end region of SCO6731, respectively. The SCO6788 disruption was analyzed with the following primer pairs: SCO6788_F and EZL2, and EZR1 and SCO6788_R (Table 2).

For constructing the *S. coelicolor SCO6731*^−^*SCO6788*^−^ double mutant, SMB07 (Am^R^) was utilized as the acceptor strain in the conjugation experiment. First, the Am resistance gene cassette of cosmid 8A04.1.h08 was replaced by the Hyg resistance gene cassette of pQM5066 by lambda-red mediated recombination. After conjugal transfer of cosmid 8A04.1.h08 (Hyg^R^) to SMB07, three independent Am^R^ Hyg^R^ Km^S^ exconjugants were isolated and checked by PCR with the primer pairs described above for mutations in SCO6731 and SCO6788. *S. coelicolor* SM09 strain was constructed by introducing the pSM01 plasmid (Table 1) into *S. coelicolor* M145 by conjugation. Following a single recombination event, the plasmid is integrated into *S. coelicolor* chromosome, yielding Spec^R^ Thio^R^ exconjugants. Clones containing a second recombination event (Spec^R^ Thio^S^) were isolated by replica plating. Three independent clones were checked by PCR using primers SCO6026_F, SCO6026_R, SCO6027_F, and SCO6027_R (Table 2).

### Plasmid construction

All plasmids used in this work are described in Table 1. To construct plasmids pSM08, pSM09 and pSM10, genes SCO6027, SCO6026, SCO6731, SCO6732, SCO6788, and SCO6789 were amplified by PCR using the following pair of primers: SCO6731_F and SCO6731_R, SCO6732_F and SCO6732_R, SCO6788_F and SCO6788_R, SCO6789_F and SCO6789_R, SCO6027_F and SCO6027_R, SCO6026_F and SCO6026_R (Table 2). PCR products were cloned in pCR-Blunt II-TOPO (Invitrogen) and submitted for DNA sequencing (University of Maine DNA sequencing facility, USA). Each individual gene was then cloned as an *Nde*I/*Eco*RI restriction fragment into pET28a yielding plasmids pSM02, pSM03, pSM04, pSM05, pSM06, and pSM07, respectively. Plasmids pSM03, pSM05, and pSM07 where then digested with *Xba*I/*Hind*III to obtain fragments harboring corresponding genes and RBS from vector. These fragments were then *Xba*I/*Hind*III cloned into pSM02, pSM04, and pSM06 vectors, respectively, yielding pSM08, pSM09, and pSM10 plasmids, which carry SCO6731-SCO6732, SCO6788-SCO6789, and SCO6027-SCO6026 operons, respectively, under the control of a T7 promoter.

For pSM01 construction, 2.5 kb regions located upstream SCO6026 (Ups_6026 fragment) and downstream SCO6027 (Dns_6027 fragment) were amplified by PCR using *S. coelicolor* genomic DNA and primers Ups_6026_F, Ups_6026_R, Dns_6027_F, and Dns_6027_R, respectively (Table 2). Spec resistance gene cassette was amplified from pIJ699 using primers Spec_F and Spec_R. PCR products where cloned in pCR-Blunt II-TOPO and checked by DNA sequencing. Spec cassette was then obtained by *Bam*HI digestion, and Ups_6026 and Dns_6027 fragments were obtained by *Hind*III/*Bam*HI and *Bam*HI/*Eco*RI digestions, respectively. The three fragments were cloned into pSET151, yielding plasmid pSM01.

### Immunoblotting

SDS-PAGE and Western Blot were carried out using standard protocols (Sambrok and Fritsch, 1989). For detection of His-tagged proteins, mouse monoclonal anti-His antibodies (QIAGEN™) were used as primary antibody and anti-mouse IgG-alkaline phosphatase conjugates were used as secondary antibody. His-tagged proteins were visualized by immunoblots using chromogenic detection as described by the manufacturer.

### Complementation assays

Competent *E. coli* Δ*fadBA* cells were transformed with plasmids pSM08, pSM09, or pSM10. The three derivatives strains were grown in M9 0.1% w/v glucose to check protein expression by Western Blot.

In order to assay the ability of the complemented strains to grow on solid and liquid M9 supplemented with lauric or oleic acid as sole carbon source, cells were cultured overnight at 37°C in liquid LB containing Kn, then diluted 1:100 in M9 medium supplemented with 0.1% w/v glucose and incubated 7 h at 25°C. At this point, samples were taken for plating on solid medium experiments (see below), and cells were harvested by centrifugation for 15 min at 4,000 × g, washed and diluted to OD_600_ 0.01 in fresh M9 supplemented with 0.1% w/v oleic acid or 0.1% w/v lauric acid. Cultures were incubated at 25°C and OD_600_measurements were carried out at different time points to evaluate growth. This experiment was performed in triplicate.

To evaluate growth on solid medium, aliquots of 3 μl OD_600_ 2.5 were taken from liquid cultures, as described above, plated on M9 supplemented with 0.1% w/v oleic acid or 0.1% w/v lauric acid and incubated at 25°C. *E. coli* BL21 and *E. coli* BL21Δ*fadBA* transformed with pET28a vector were used as controls.

### Reporter fusion construction

To evaluate the expression profile of the SCO*fadBA* genes in liquid media, plasmids pSM14, pSM15, and pSM16 were constructed. These vectors are integrative in *Streptomyces* and contain the *xylTE* genes under the transcriptional control of the SCO6730, SCO6785, and SCO6027 promoter region, respectively (Table 1). The putative promoter regions were amplified by PCR using *S. coelicolor* genomic DNA as template and primers P_SCO6730_F, P_SCO6730_R, P_SCO6785_F, P_SCO6785_R, P_SCO6027_F, and P_SCO6027_R (Table 2). Each primer pair was designed to yield a 223 bp amplicon that extends from −210 to +13 relative to the start codon of each gene. PCR products were cloned in a pCR-TOPO-Blunt vector and the integrity of each sequence was confirmed by DNA sequencing. Promoters DNA fragments were obtained by restriction with *Bam*HI/*Eco*RI and cloned into the pCA75 vector; yielding plasmids pSM14, pSM15, and pSM16. These intergenic regions were selected for amplification based on *in silico* analysis and on previous transcription start site (TSS) mapping (Jeong et al., 2016).

Expression patterns were also analyzed in solid medium; to this purpose pSM17, pSM18, and pSM19 plasmids were constructed. In this case, PCR fragments corresponding to the promoter regions under study were cloned into the pCA73 vector generating *cbg* transcriptional fusions (Table 1).

Finally, plasmid containing each reporter fusion was introduced into *S. coelicolor* M145 by conjugation using *E. coli* ET12567/pKOS111-47 as donor, yielding the reporter strains SMB01, SMB02, SMB03, SMB04, SMB05, and SMB06 (Table 1).

### Assay of catechol 2,3-dioxygenase in cell free extracts

Strains SMB01, SMB02, and SMB03 (Table 1) were cultured in SMM minimal medium supplemented with different carbon sources (1% (w/v) glucose, 1% (w/v) glucose plus 0.1% (w/v) oleic acid or 0.1% (w/v) oleic acid) at 30°C for 15 and 40 h. Cells were then harvested by centrifugation at 5,000 × g during 5 min, washed with a buffer solution containing 50 mM Tris-HCl pH 8 and stored at −20°C for further processing. Catechol 2,3-dioxygenase activity was then measured as indicated in (Kieser et al., 2000). Protein concentration was determined with Bradford reagent (Bradford, 1976). Catechol 2,3-dioxygenase activities were normalized to protein concentration.

### Assay of luciferase activity in solid medium

Strains SMB04, SMB05, and SMB06 (Table 1) were cultivated in solid SMM medium supplemented with luciferin and different carbon sources. This assay was carried out in a white opaque 96-well plate. Each well was filled with 180 μl solid SMM medium supplemented with 10 mM luciferin (Sigma Aldrich) and the corresponding carbon source: 0.2% (w/v) glucose, 0.2% (w/v) glucose plus 0.2% (w/v) oleic acid, or 0.2% (w/v) oleic acid. Each well was inoculated whit 3,000 spores of each strain. Six replicates were made for each strain in every condition. Plates were then incubated at 30°C during 70 h in a Synergy® spectrophotometer, programmed to measure luminescence emission every 30 min.

The luminescence intensity registered in every well-depends on two factors: (1) the activity of the promoter fused to the *cbg* gene in a particular medium; and (2) the amount of cells present in the well at the time of the measurement; more cells will produce a larger emission. Since *Streptomyces* shows different growth rates according to the carbon source present in the medium, the data obtained from this experiment were normalized using a P_*hrdB* (*hrdB* promoter)*-cbg* transcriptional fusion. For this purpose, a *S. coelicolor* strain carrying a transcriptional fusion to the *hrdB* promoter was grown under the same conditions as the reporter strains and luminescence measurements were made at the same time points.

### Chemostat cultures

For the continuous fermentations of *S. coelicolor* the wild type strain M145 was used. The inoculum was prepared following a two-step procedure. A 20% (w/v) glycerol spore aliquot prepared as described above was used to inoculate a 500 ml Erlenmeyer flask containing 100 ml of GG1 medium (1.5% (w/v) soy peptone; 0.3% (w/v) NaCl; 0.1% (w/v) CaCO_3_; 1.5% (w/v) glucose; 1.2% (v/v) glycerol) to reach an initial concentration of 10^6^ spores/ml. Dispersed growth was achieved by the use of a magnetic stirrer and a triangular magnetic bar. After incubation at 30°C for 48 h, 10 ml of the culture were transferred to a 500 ml Erlenmeyer containing 100 ml of GYB medium (3.3% (w/v) glucose; 1.5% (w/v) yeast extract) and incubated at 30°C for 24 h. Subsequently, a 1.5 l bioreactor (Adaptive Biosystems 7000, UK) containing 950 ml of Modified Evans medium (0.62% (w/v) NaH_2_PO_4_.2H_2_O; 0.075% (w/v) KCl; 0.025% (w/v) MgCl_2_.6H_2_O; 0.028% (w/v) (NH_4_)_2_SO_4_; 0.0037% (w/v) CaCl_2_.2H_2_O; 0.038% (w/v) citric acid; 0.02% (v/v) Breox antifoam; 0.133% (w/v) NaOH; 0.1% (v/v) trace elements) with an initial glucose concentration of 9 g/L was inoculated with 50 ml of the GYB inoculum. An initial batch phase of ~24 h was allowed to elapse until the initial glucose was completely depleted. At that time, fresh medium was fed to maintain a constant dilution rate of 0.039 h^−1^. The culture was kept at a constant volume of 1 L. Foaming was prevented by manually addition of a suspension of 10% (v/v) antifoam agent (BDH Laboratory Supplies, UK). The pH was monitored and controlled at 6.8 ± 0.2 by the automatic addition of 2 N NaOH or 2 N HCl. The temperature was kept at 30°C, the stirring speed was set at 1,000 rpm and the air flow rate at 1.25 l/min. The cells grew in dispersed form (as assessed by microscopic observation) throughout the fermentation period. During the process, the concentrations of biomass, glucose, actinorhodin, ${\text{NH}}_{4}^{+}$, ${\text{PO}}_{4}^{3-}$, and pyruvate in the supernatant were measured, as well as the content of total lipids and TAG in the biomass, and the O_2_ and CO_2_ content in the outcoming gases. From those parameters, consumption, and consumption rates were calculated. Based on the values, it was determined that steady state was reached after seven volume changes in the bioreactor. The total duration of these fermentations was 180 h.

### Flux balance analysis

The analysis of metabolic models through FBA was performed using the COBRA Toolbox (Schellenberger et al., 2011) framework (opencobra.github.io) operating in the MATLAB R2014b software environment (mathworks.com/products/matlab/). All calculations were carried out on a personal computer with OSX operating system El Capitan v.10.11.2 with an Intel® Core i5 2.70 GHz 8 GB 1867 MHz DDR3 processor.

### Lipid analysis

Total lipids of the *S. coelicolor* strains were extracted twice from lyophilized cell material (2 mg) with chloroform/methanol (2:1 v/v). The combined extracts were evaporated and analyzed by TLC on silica gel 60 F254 plates (0 ± 2 mm, Merck) as described previously (Wältermann et al., 2000), using the solvent mixture hexane/diethylether/acetic acid (80:20:1, v/v/v). Lipid fractions were visualized by Cu-phosphoric staining. For radioactively labeled samples, lipids were visualized by autoradiography using a Storm 840 PhosphorImager (Amersham Biosciences®). For densitometry analysis, images where digitalized and analyzed using the software ImageJ v1.48.

### [^14^C]-acetate incorporation assay

*S. coelicolor* M145, SMB07, SMB08, and SMB09 strains were cultured in minimal SMM medium supplemented with 0.2% (w/v) glucose at 30°C. After 15, 40, and 90 h of incubation, 3 ml samples were taken from each culture and incubated during 1 h with 2 μCi [^14^C]-acetate. Cells were then harvested by centrifugation at 5,000 × g for 10 min at 4°C and washed three times with a buffer containing Tris-HCl 50 mM pH 8.0. Total lipids were then extracted following the standard protocol (Folch et al., 1957), resolved by TLC and visualized by autoradiography as previously described.

### Pulse and chase assay

*S. coelicolor* M145, SMB07, SMB08, and SMB09 strains were cultured in minimal SMM medium supplemented with 0.2% (w/v) glucose at 30°C. After 10 h, 2 μCi [^14^C]-acetate was added and cultures were further incubated for 10 h. Cells were then harvested by centrifugation at 5,000 × g for 10 min at 4°C and washed three times with a buffer containing Tris-HCl 50 mM pH 8.0. Cells were resuspended in fresh SMM medium without a carbon source and incubated at 30°C during 70 h. Samples of each culture were harvested at different times by centrifugation at 5,000 × g for 10 min at 4°C and cells washed three times with a buffer containing Tris-HCl 50 mM pH 8.0. Total lipids were then extracted and processed as described above.

### Triacylglycerol quantification by high resolution LC-MS

For quantification of triacylglycerides, the biomass was lyophilized and weighed, and 1 mg was processed for total lipid extraction as described in Bligh and Dyer (1959). Ten micrograms of cetylpalmitate was added before extraction as an internal standard for load control. Then 500 μL of chloroform were added to solubilize lipids and 50 μL of the lipid extract were diluted in an equal part of methanol. Five microliters of this solution were injected and separated on a ZORBAX Eclipse XDB-C8 column (3.0 × 50 mm, particle size = 1.8 μm; Agilent, USA) using methanol supplemented with 5 mM ammonium acetate as the mobile phase. The outlet of the liquid chromatograph was connected to a micrOTOF mass spectrometer (Bruker Daltonik, Bremen, Germany) operating in the positive-ion mode, and the data was acquired online in the mass range m/z 300–1,500. TAG were detected as ammonium adducts in the range of 6–20 min of the chromatography run. A calibration curve was done using pure tripalmitin as a standard. The TAG concentration in the samples was calculated by the linear regression equation obtained from the calibration curve.

### Analysis of FA composition of tag by GC-MS

Fatty acid analysis of TAG inclusions was done by preparing FA methyl esters by transesterification of isolated lipid bodies with 0.5 M sodium methoxide in methanol and analysis with a Perkin-Elmer Turbo Mass gas chromatograph-mass spectrometer on a capillary column (30 m by 0.25 mm internal diameter) of 100% of dimethylpolysiloxane (PE-1; Perkin-Elmer, Waltham, MA). Helium at 1 ml min^−1^ was used as the carrier gas, and the column was programmed at 4°C min^−1^ from 140 to 240°C. Branched-chain FAs, straight-chain FAs, and unsaturated FAs used as reference compounds were obtained from Sigma-Aldrich (Sigma Aldrich, St Louis, MO).

## Results

### Identification of putative *fadAB* genes in *S. coelicolor*

**Figure 1:**
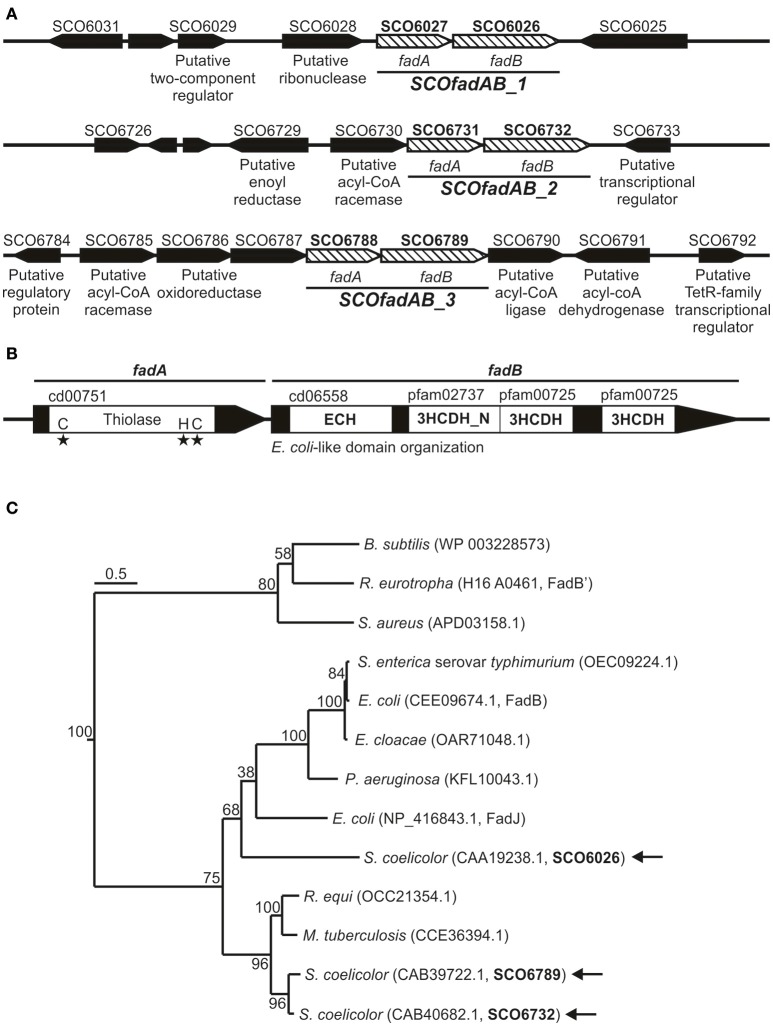
Gene clusters coding for putative FadAB complexes in S. coelicolor. **(A)** Schematic representation of the genome context of the *S. coelicolor* clusters SCO6027-SCO6026, SCO6731-SCO6732, and SCO6788-SCO6789. Gene names or their putative encoding activities are given below the arrows, which indicate the positions, relative lengths, and directions of transcription of neighboring genes. **(B)** Conserved domain organization and protein family classification of FadA and FadB enzymes. **(C)** Neighbor-joining phylogenetic tree based on amino acid sequence of representative and characterized FadB enzymes. Sequence alignment and tree construction were made using open access software SeaView 4 (Gouy et al., 2010). Bootstrap values (100 replicas) are shown along the branches. The scale bar represents substitutions per site. Protein IDs are given in parentheses after the names of their source organisms. Arrows indicate the three S. coelicolor FadB studied in this work.

In order to identify β-oxidation coding genes in *S. coelicolor*, we carried out a homology search using as query the amino acid sequences of the *fadA, fadB*, and *fadE* gene products from *E. coli*. Our search showed that the *S. coelicolor* genome contains 9 ORF homologs to FadA (ID 948324), 3 ORF homologs to FadB (ID 948336), and 14 ORF exhibiting moderate homology to FadE (ID 949007). Each of the three putative *fadB* genes (SCO6026, SCO6732, and SCO6789) were located adjacent to a putative *fadA* (SCO6027, SCO6731, and SCO6788), suggesting possible co-transcriptional units (Figure 1A). Only these three gene clusters -SCO6027-SCO6026, SCO6731-SCO6732, and SCO6788-SCO6789- possess orthologs and exhibit a notable synteny with other sequenced *Streptomyces* species, such as *S. scabiei, S. avermitilis, S. flavogriseus, S. griseus*, and *S. cattleya*. Interestingly, proteins encoded by clusters SCO6731-SCO6732 and SCO6788-SCO6789 exhibit percentages of identity above 85% and similarity above 90%, which might suggest that one of them resulted from a genetic duplication event. Also, these two clusters are surrounded by putative FA metabolism-related genes (a feature not observed for cluster SCO6027-SCO6026) (Figure 1A).

On the other hand, the large redundancy in *fadE* genes difficult the assignment of possible candidates involved in FA catabolism.

All FadA candidates show the conserved thiolase domain (cd00751) as well as the three conserved active site residues that define this activity (Figure 1B). Similarly, all three possible FadB exhibit the same so-called *E. coli*-like structure of 3-hydroxyacyl-CoA dehydrogenases (Figure 1B; Volodina and Steinbüchel, 2014). This organization consists of an N-terminal crotonase-like family/enoyl-CoA hydratase domain (cd06558/cl23717), a central 3-hydroxyacyl-CoA dehydrogenase-NAD binding domain (3HCDH_N, Pfam 02737) and one or two 3-hydroxyacyl-CoA dehydrogenase domains (3HCDH, Pfam 00725) located at the C-terminal portion of the protein (Figure 1B).

Neighbor-joining phylogenetic trees based on amino acid sequence of FadB enzymes from different organisms, group SCO6732 and SCO6789 together with FadB from the actinomycetes *Mycobacterium tuberculosis* (64 and 63% identity, respectively; Srivastava et al., 2015) and *Rhodococcus equi* (64 and 61% identity, respectively; Kelly et al., 2002; Figure 1C). In contrast, SCO6026 is placed near to FadB sequences from Gram (−) bacteria: *E*. *coli* FadJ (30% identity), *Pseudomonas aeruginosa* FadB (30% identity), *Enterobacter cloacae* FadB (28% identity), *E. coli* FadB (28% identity), and *Salmonella enterica* serovar Typhimurium FadB (28% identity). On the other hand, *Bacillus subtilis* FadB (Matsuoka et al., 2007), *Ralstonia eutropha* FadB' (Volodina and Steinbüchel, 2014), and *Staphylococcus aureus* FadB (Khairon et al., 2016) group together and, unlike the other FadB considered, exhibit a *Burkholderia*-like domain organization, which consist in an inverted order of domains compared with *E. coli* [3-hydroxyacyl-CoA dehydrogenases domains located at the N-terminus Volodina and Steinbüchel, 2014].

Overall, the *in silico* analysis suggested that the three putative *fadAB* homologues identified in *S. coelicolor* were probable candidates to code for β-oxidation enzymes. Gene clusters SCO6027-SCO6026, SCO6731-SCO6732, and SCO6788-SCO6789 are referred hereafter as *SCOfadAB_1, SCOfadAB_2*, and *SCOfadAB_3*, respectively.

### Growth complementation of an *E. coli* Δ*fadBA* mutant with *S. coelicolor* FadAB proteins

**Figure 2:**
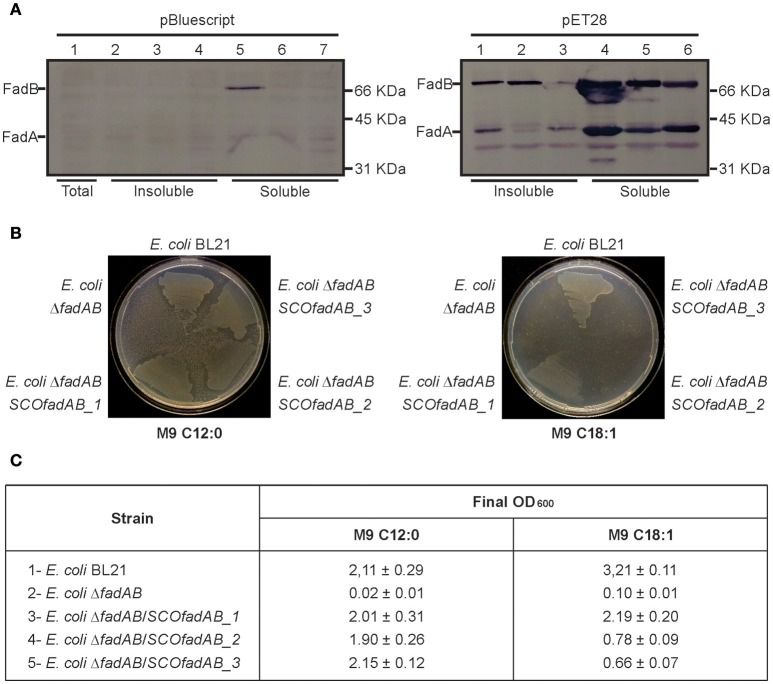
Complementation of *E. coli* Δ*fadBA* mutant strain with the *SCOfadAB* proteins. **(A)** Immunoblot of crude extracts of *E. coli* Δ*fadBA* expressing *SCOfadAB* proteins from two different expression systems in the absence of inducer. Gel 1 (left): Lane 1: *E*. *coli* Δ*fadBA*:pBluescript II SK (total crude extract); Lane 2: *E*. *coli* Δ*fadBA*:pSM11 (insoluble fraction); Lane 3: *E*. *coli* Δ*fadBA*:pSM12 (insoluble fraction); Lane 4: *E*. *coli* Δ*fadBA*:pSM13 (insoluble fraction); Lane 5: *E*. *coli* Δ*fadBA*:pSM11 (soluble fraction); Lane 6: *E*. *coli* Δ*fadBA*:pSM12 (soluble fraction); Lane 7: *E*. *coli* Δ*fadBA*:pSM13 (soluble fraction). Gel 2 (right): Lane 1: *E*. *coli* Δ*fadBA*:pSM08 (insoluble fraction); Lane 3: *E*. *coli* Δ*fadBA*:pSM09 (insoluble fraction); Lane 4: *E*. *coli* Δ*fadBA*:pSM10 (insoluble fraction); Lane 5: *E*. *coli* Δ*fadBA*:pSM08 (soluble fraction); Lane 6: *E*. *coli* Δ*fadBA*:pSM09 (soluble fraction); Lane 7: *E*. *coli* Δ*fadBA*:pSM10 (soluble fraction). **(B)** Complementation studies to monitor the growth of *E. coli* BL21, *E. coli* Δ*fadBA* and the *E. coli* Δ*fadBA* strains expressing the three different set of *SCOfadAB* genes in different media. M9 minimal medium plates were supplemented with 0.2% of the specified fatty acids. C12:0 and C18:1 plates were incubated for 7 and 11 days, respectively. The clear zones around the areas of growth are due to consumption of lauric acid (C12:0), which produces turbidity in the medium due to its partial insolubility. **(C)** Growth of each strain on liquid M9 minimal medium supplemented with C12:0 or C18:1 and final OD_600_ reached after 3- and 6-days of incubation, respectively.

In *E. coli, fadBA* gene products are required for aerobic growth on FA as sole carbon sources (Yang et al., 1988; Campbell et al., 2003). Therefore, to study *in vivo* the physiological role of the identified *SCOfadAB* clusters, we carried out *E. coli* genetic complementation tests. For this, we first constructed an *E. coli* Δ*fadBA* mutant and then individual *SCOfadAB* clusters under the T7 promoter were introduced in the resulting FA auxotrophic strain (*E. coli* Δ*fadBA*, Table 1). Western blot analysis showed that all *SCOfadAB* gene products form inclusion bodies in the corresponding *E. coli* recombinant strains; however, the expression that leaks from the T7 promoter is sufficient to obtain detectable quantities of soluble protein (Figure 2A). Therefore, complementation tests were performed at 25°C with no addition of the inducing agent isopropyl b-D-1-thiogalactopyranoside (IPTG).

Expression of the three *SCOfadAB* clusters rescued growth of *E. coli* Δ*fadBA* on M9 plates containing lauric acid (C_12:0_) as sole carbon source after 7 days (Figure 2B). In this medium, the three complemented strains displayed the same growth behavior as *E. coli* BL21; whereas *E. coli* Δ*fadBA* transformed with an empty plasmid showed no growth. In liquid M9 medium supplemented with 0.1% (w/v) lauric acid as sole carbon source, all three complemented strains reached final OD_600_-values very similar to that of the BL21 (Figure 2C), confirming the successful complementation of the *E. coli* Δ*fadBA* mutant with the three different putative FadAB enzymes from *S. coelicolor*.

When the complementation test was carried out in the presence of oleic acid (C_18:1_) as sole carbon source, the three complemented strains showed different growth phenotypes. In M9 agar containing 0.1% (w/v) of C_18:1_, *E. coli* BL21 showed visible growth after incubation for 5 days, whereas *E. coli* Δ*fadBA*/*SCOfadAB_1* exhibited significant growth after 9 days. *E. coli* Δ*fadBA*/*SCOfadAB_2* and *E. coli* Δ*fadBA*/*SCOfadAB_3* only displayed small colonies after 11 days (Figure 2B). Similar results were observed when the complementation test was carried out in liquid M9 with oleic acid as sole carbon source. In this case, all three complemented strains were able to grow, albeit at very different growth rate and reaching reduced final OD_600_ values. As shown in Figure 2B, *E. coli* BL21 reached a final OD_600_ of 3.21 ± 0.11 after incubation for 3 days, whereas complemented strains reached lower OD_600_ after 6 days. *E. coli* Δ*fadBA* transformed with an empty plasmid did not show any growth at these conditions.

Altogether, these results indicate that *SCOfadAB* gene products can display catalytic activities attributable to *E. coli* FadBA complex when expressed in the *E. coli* Δ*fadBA* mutant, namely: enoyl-CoA hydratase, 3-hydroxyacyl-CoA dehydrogenase, and 3-ketoacyl-CoA thiolase (Yang et al., 1988; Srivastava et al., 2015). Furthermore, the complementation studies also suggest that the other β-oxidation enzymes (FadL, FadD, and FadE) present in *E. coli* can functionally interact with *SCOfadAB* gene products for exogenous FA assimilation.

### *S. coelicolor fadAB* clusters exhibit different expression patterns

*S. coelicolor* possesses a complex life cycle that involves different morphological stages which are dependent on growth conditions and media composition. The morphological differentiation that occur in solid medium can lead to gene expression patterns which may differ from those taking place in liquid medium (Yagüe et al., 2013, 2014). Thus, in order to assess the expression patterns in both types of media, we constructed *S. coelicolor* M145 derivative strains containing ectopic integrations of *cbg* and *xylTE* transcriptional fusions (Table 1).

**Figure 3:**
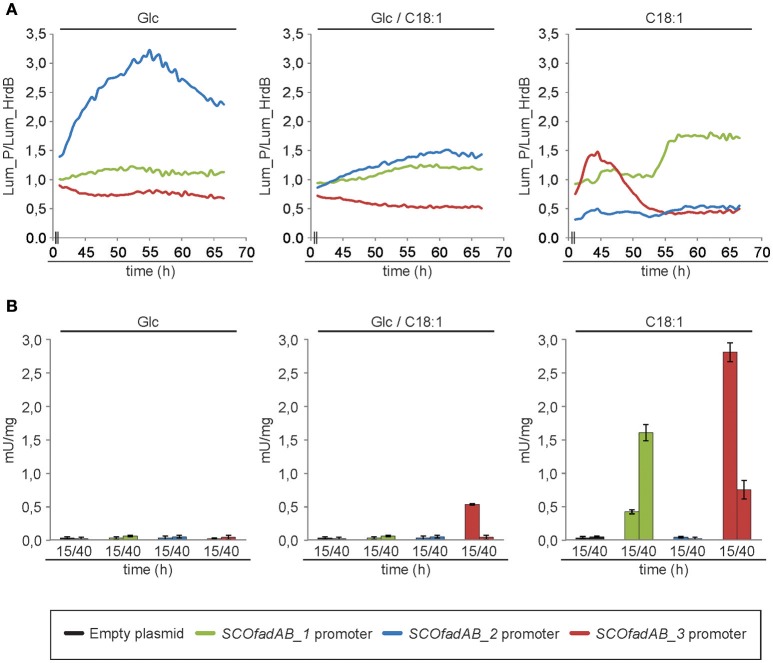
Expression profiles of the *SCOfadAB* clusters in *S. coelicolor*. **(A)** Expression profiles of the *SCOfadAB* promoters in solid SMM medium supplemented, with glucose, oleic acid or glucose plus oleic acid as sole carbon sources. Luminescence was measured throughout 70 h of incubation at 30°C and the expression profiles were normalized to the luminescence of the transcriptional fusion to *hrdB* promoter **(B)** Expression profiles in SMM liquid medium were carried out in the strains containing the *xylTE* transcriptional fusions. CDO-specific activity was determined from samples collected of bacterial cultures grown for 15 h and 40 h in SMM liquid medium at 30°C. The data are the average of three independent experiments performed in duplicate. Bars represent standard deviation.

The luciferase encoded by *cbg* gene generates visible light during luciferin oxidation. Strains of *S. coelicolor* carrying transcriptional fusions of the three different *SCOfadAB* promoters to *cbg* were grown in solid SMM medium supplemented with glucose, oleic acid or glucose plus oleic acid as carbon sources and luciferin for light emission. Cultures were incubated at 30°C for 70 h and the expression profiles were normalized using as reference the luminescence emission of *cbg* transcriptional fusion to *hrdB* promoter. The *hrdB* product is a vegetative σ factor whose expression is constitutive and independent of the carbon source (Buttner et al., 1990; Shiina et al., 1991; Marcos et al., 1995). Under these conditions, expression from *SCOfadAB_1* and *SCOfadAB_3* promoters was markedly enhanced when oleic acid was the sole carbon source, although at different growth times (Figure 3A). *SCOfadAB_1* promoter exhibited maximum luminescence emission after 55 h (late culture times), whereas *SCOfadAB_3* did it until 50 h (early culture times). Also, both promoters led to constitutive *cbg* expression when glucose was present in the medium. In contrast, *SCOfadAB_2* promoter showed maximum expression at 55 h when glucose was the sole carbon source, while its expression was constitutive, although at considerable lower levels, when oleic acid was present in the medium (Figure 3A).

To evaluate the expression patterns of the *SCOfadAB* promoters in liquid media, the strains containing each of the three *xylTE* transcriptional fusions [*Pseudomonas putida* genes coding for catechol 2,3-dioxygenase (CDO; González-Cerón et al., 2001] were grown in SMM medium supplemented with glucose, oleic acid, or glucose plus oleic acid as carbon sources and the CDO activity measured in cell free extracts from samples taken at 15 and 40 h (exponential and late-stationary phases of growth, respectively). The expression from *SCOfadAB_1* and *SCOfadAB_3* promoters was detectable only in the presence of oleic acid (Figure 3B). In agreement with the results obtained in solid media, expression from *SCOfadAB_1* promoter was higher at late stages of growth (stationary phase), whereas expression from *SCOfadAB_3* promoter was higher at early stages of growth (exponential phase). In contrast, no CDO activity was detected from the *SCOfadAB_2* promoter in any of the media tested.

Taken together, the expression profiles obtained with the transcriptional fusions studies strongly suggest that *SCOfadAB_1* and *SCOfadAB_3* might have a role in exogenous FA assimilation at different growth stages. These results also indicate that the three clusters are differentially expressed in *S. coelicolor* depending on growth stage and media composition.

### Prediction of mutations that would increase tag accumulation

To assess the role of β-oxidation genes in TAG accumulation and to identify other mutations that could lead to an increased TAG pool, we performed FBA using a *S. coelicolor* genome-scale metabolic network.

Recently, a genome-scale metabolic model named iMK1208 was published for *S. coelicolor* A3(2) (Kim et al., 2014). Although it is a high-quality metabolic reconstruction, TAG biosynthesis in the model is restricted to FAS intermediates as it only considers TAG production from DAG and acyl-ACP (Kim et al., 2014). However, the three principal WS/DGAT involved in TAG biosynthesis in *S. coelicolor* use long-chain acyl-CoA as acyl donors (Arabolaza et al., 2008). Therefore, we introduced reactions in iMK1208 in which TAG is biosynthesized from DAG and acyl-CoA without modification of the biomass equation. This version of iMK1208 is referred to as m_iMK1208 (modified_iMK1208, Supplementary Material).

In order to obtain experimental values to constrain *in silico* simulations, we carried out chemostat cultures of *S. coelicolor* wild type strain at a dilution rate of 0.039 h^−1^. To validate the modified model, we performed FBA simulations with both iMK1208 and m_iMK1208 models. Using as constraints the experimental values obtained in the chemostat experiments, we predicted growth rate values of 0.042 h^−1^; which demonstrated the validity and consistency of the modified metabolic model m_iMK1208. We then used the computational OptKnock framework (Burgard et al., 2003) in order to predict possible mutations leading to an increase in carbon fluxes toward TAG biosynthesis. OptKnock was performed with both models with the output set to obtain 10 candidate reactions (Table 3). Simulations using iMK1208 identified the inactivation of reactions involved in actinorhodin biosynthesis (ACTS18), phospholipid precursors biosynthesis (ACPPAT160, AGPATi140, AGPATi160, APG3PATai150, and CLPNS180), degradation of Thr to Gly (GLYAT and THRD) and glycolysis (FBA and PFK). On the other hand, simulations based on m_iMK1208 identified the inactivation of reactions involved in actinorhodin biosynthesis (ACTS18), acyl-CoA degradation (FACOAE80), synthesis of phospholipid precursors (ACPPAT181, APH120, APH141, and GPDDA3e), and notably, β-oxidation of FA [ACACT2r (FadA), ACACT8r (FadA), ACOAD6f (FadE), and ECOAH8 (FadB)]. This output further supports m_iMK1208 as a consistent model for predictions related to neutral lipid metabolism and validated the construction of knockout mutant strains for the identified *SCOfadAB* gene clusters.

**Table 3:** Optknock predicted enzyme reactions to knockout in order to increase carbon flux toward TAG biosynthesis.

**Reaction**	**Proposed enzyme**	**Putative genes**	**Related metabolism**
**iMK1208-BASED PREDICTION**			
ACTS18	Hydroxylated dihydrokalafungin thioesterase		Actinorhodin biosynthesis
ACPPAT160	acyl-(acyl carrier protein):phosphate acyltransferase (C16:0)		Glycerophospholipid metabolism
AGPATi140	1-Tetradecanoyl-sn-glycerol 3-phosphate O-acyltransferase (iso-C14:0)	SCO0920 SCO1085 SCO1228 SCO1566 SCO1759 SCO2122	
AGPATi160	1-Hexadecanoyl-sn-glycerol 3-phosphate O-acyltransferase (iso-C16:0)	SCO0920 SCO1085 SCO1228 SCO1566 SCO1759 SCO2122	
APG3PATai150	Acyl-phosphate:glycerol-3-phosphate acyltransferase (ante-iso-C15:0)		
CLPNS180	Cardiolipin synthase (n-C18:0)	SCO7081	
GLYAT	Glycine C-acetyltransferase	SCO6800	Glycine and serine metabolism
THRD	L-threonine dehydrogenase	SCO6799	
FBA	Fructose-bisphosphate aldolase	SCO3649	Glycolysis
PFK	Phosphofructokinase	SCO5426 SCO2119 SCO1214	
**m_iMK1208-BASED PREDICTION**			
ACTS18	Hydroxylated dihydrokalafungin thioesterase		Actinorhodin biosynthesis
FACOAE80	Fatty-acid-CoA thioesterase (octanoate)	SCO1153 SCO2773	Cell envelope biosynthesis
ACPPAT181	Acyl-(acyl carrier protein):phosphate acyltransferase (C18:1)		Glycerophospholipid metabolism
APH120	Acylphosphatase (C12:0)	SCO5576	
APH141	Acylphosphatase (C14:1)	SCO5576	
GPDDA3e	Glycerophosphodiester phosphodiesterase (Glycerophosphoserine)	SCO1090 SCO1419 SCO3976 SCO5661	
ACACT2r	Acetyl-CoA C-acyltransferase (butanoyl-CoA) (r)	**SCO6788** SCO1324 SCO4502 **SCO6027**	Fatty acid metabolism
ACACT8r	Acetyl-CoA acyltransferase (hexadecanoyl-CoA) (r)	**SCO6788** SCO1324 SCO4502 **SCO6027**	
ACOAD6f	Acyl-CoA dehydrogenase (tetradecanoyl-CoA)	SCO1690 SCO2774 SCO6787	
ECOAH8	3-Hydroxyacyl-CoA dehydratase (3-hydroxyoctadecanoyl-CoA)	**SCO6732** SCO4384	

*In bold are the genes addressed in this study that are also predicted by simulation whit m_iMK1208*.

### Inactivation of *S. Coelicolor fadAB* clusters reduces tag mobilization

To assess the physiological consequences of inactivating *SCOfadAB* gene clusters, we constructed single and double mutant strains, in which one or two of the clusters were knocked out (See Table 1).

All single and double mutants, and the wild type *S. coelicolor* M145 strain, were able to grow on minimal medium supplemented with FA of different chain length as sole carbon sources. Similarly, all six mutants displayed no differences with M145 in terms of growth rate, morphological differentiation and antibiotic production in rich medium.

**Figure 4:**
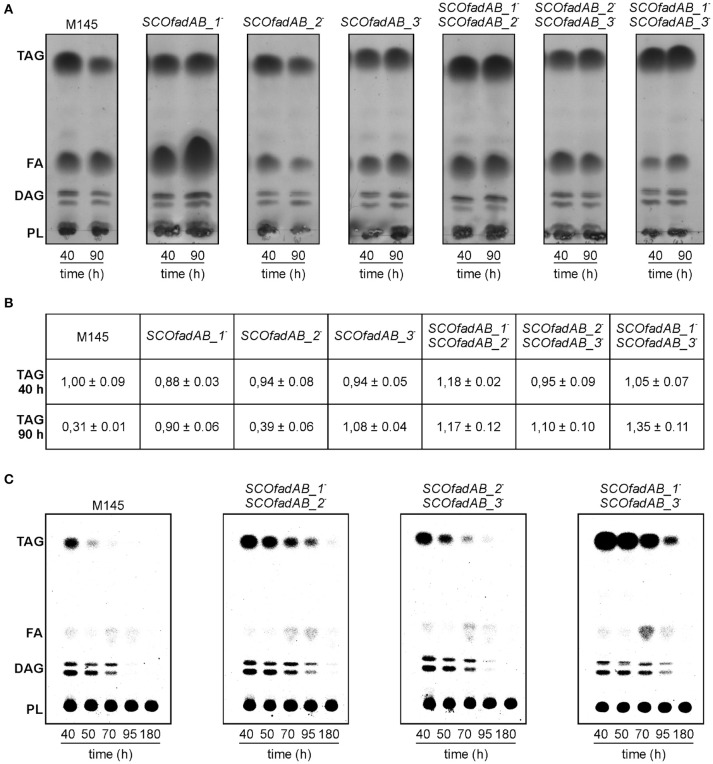
Analysis of lipid composition in *S. coelicolor* single and double *SCOfadAB* knockout mutants. **(A)** Total lipids extracted from 2.5 mg of lyophilized cells obtained from samples at 40 and 90 h of R5 cultures of the indicated strains were analyzed on silica gel TLC plates developed in hexane/diethylether/acetic acid (80:20:1, v/v/v), using Cu-phosphoric solution as visualization reagent. **(B)** Relative TAG content of the wild type, single and double mutant strains analyzed in **(A)**. Three independent total lipids TLC analysis were digitalized for each strain and the spots were quantified using ImageJ v1.48 software. The densitometry value obtained for TAG content of M145 strain at 40 h of growth was assigned the value of 1. **(C)** Pulse-chase analysis of TAG after switching cultures to a SMM minimal medium without carbon source. Cell samples were collected immediately after medium shift (40 h) and subsequently, at the indicated time points. Total lipids were extracted from 2.5 mg of lyophilized [^14^C]-acetic acid-pulse labeled cells of each *S. coelicolor* double mutant strains and analyzed as described for **(A)**. Radiolabeled lipid species were visualized using a PhosphoImager Screen.

However, all mutant strains exhibited a notable increase in TAG accumulation compared with the wild type strain M145 when grown until late stationary phase in both rich and minimal media. The lipid composition of *S. coelicolor* wild type and single *SCOfadAB* knockout mutants at 40 and 90 h was determined by TLC. In rich medium, the single mutants *SCOfadAB_1*^−^ and *SCOfadAB_3*^−^ showed an increased amount of accumulated TAG of 2.9 and 3.5-fold, respectively, at late stationary phase (90 h), compared to the M145 parental strain (Figure 4A). The single mutant *SCOfadAB_2*^−^ showed a small difference in TAG accumulation compared with M145 (1.3-fold increase, Figures 4A,B). Similar results were observed when these strains were grown in minimal medium, which is shown in Supplementary Figure 1.

Accordingly, TAG accumulation in the double mutant *SCOfadAB_2*^−^*SCOfadAB_3*^−^ was similar to that observed for single mutant *SCOfadAB_3*^−^. However, the double mutant *SCOfadAB_1*^−^*SCOfadAB_2*^−^ showed a TAG content 3.8-fold higher than M145, which represents an increase higher than that observed for each corresponding single mutant. The same was observed for the double mutant *SCOfadAB_1*^−^*SCOfadAB_3*^−^, which exhibited TAG levels 4.3-fold higher than the M145 strain (Figures 4A,B). In these conditions (90 h in rich medium), M145 accumulates 11.2 ± 0.02 μg TAG/mg DCW, equivalent to 1.1% of the dry cell weight; whereas double mutant *SCOfadAB_1*^−^*SCOfadAB_3*^−^ accumulates 48.12 ± 0.03 μg TAG/mg DCW, equivalent to 4.8% of the dry cell weight. The FA composition of the TAG accumulated for each of these two strains is shown in Supplementary Figure 4.

The highest levels of TAG found in the double mutant strains *SCOfadAB_1*^−^*SCOfadAB_2*^−^ and *SCOfadAB_1*^−^*SCOfadAB_3*^−^ suggest that all three clusters could be involved in the degradation of FA derived from TAG mobilization. In order to confirm this hypothesis, we carried out [^14^C]-acetate incorporation assays and pulse chase experiments. [^14^C]-acetate incorporation occurs to the same extent in M145, single, and double mutant strains at both exponential and late stationary phase (data not shown). These results suggest that the increased TAG content in the mutant strains is not due to an increased TAG biosynthesis rate.

Pulse and chase experiments consisted in monitoring a [^14^C]-labeled pool of TAG after switching cultures to a minimal medium with no carbon source, conditions that are favorable for TAG mobilization (Figure 4C). Strain M145 showed a typical decrease in TAG content after the culture reached late stationary phase. In contrast, all three double mutant strains exhibited decreased TAG mobilization, although to different extents. The relative TAG and FFA content of the wild type and double mutant strains analyzed in the pulse-chase experiment shown in Figure 4C is presented in Supplementary Figure 2.

The double mutant *SCOfadAB_2*^−^*SCOfadAB_3*^−^ showed TAG mobilization close to that observed in the wild type; however, a significant content of TAG is still observed after 70 h in the absence of carbon source, in contrast to M145, which showed a completely depleted pool at this time. The double mutant *SCOfadAB_1*^−^*SCOfadAB_2*^−^ mobilized TAG to a lesser extent than the double mutant *SCOfadAB_2*^−^*SCOfadAB_3*^−^ and displays a significant TAG content after 95 h of culture in absence of carbon source. Remarkably, the strongest TAG over-accumulating phenotype is exhibited by double mutant *SCOfadAB_1*^−^*SCOfadAB_3*^−^, which showed a significant TAG content until 95 h of culture in the absence of carbon source. This is in agreement with the total lipid content observed in rich medium (Figure 4A) and with the pulse and chase experiments carried out with single mutant strains (Supplementary Figure 3). Overall, these findings strongly suggest that all three identified *SCOfadAB* gene clusters are involved in FA β-oxidation in *S. coelicolor*.

## Discussion

Microbial TAG accumulation has captured much attention since it offers a potential source for renewable oleochemicals (Lennen and Pfleger, 2013; Alvarez, 2016; Ledesma-Amaro and Nicaud, 2016). Although TAG biosynthetic routes in *Streptomyces* have been addressed in various studies (Hobbs et al., 1997; Arabolaza et al., 2008; Rodriguez et al., 2012; Röttig et al., 2016a,b), the metabolic pathways for TAG catabolism remain poorly explored. At late stationary phase in submerged liquid culture, TAG mobilization is followed by β-oxidation of the released FA (Olukoshi and Packter, 1994). Thus, it seems possible that β-oxidation would play a central role in the regulation of TAG mobilization and, therefore, in TAG accumulation. However, genes directly involved in this metabolic pathway had not been identified so far.

This work represents the first study of genes/proteins involved in the β-oxidation pathway in *Streptomyces*. By carrying out a homology search we identified 14 putative FadE, 9 putative FadA, and 3 putative FadB encoding genes. A high degree of gene redundancy is a common feature in actinomycetes. For example, *M. tuberculosis* possesses 35 transcriptionally active *fadE* genes, which are believed to be involved in the catabolism of structurally diverse FA during growth on different environments (Schnappinger et al., 2003). Here, we focused on characterizing the three putative FadAB coding clusters: SCO6027-SCO6026, SCO6731-SCO6732, and SCO6788-SCO6789. The three sets of *SCOfadAB* genes were able to complement growth of an *E. coli* Δ*fadAB* unable to grow in saturated (lauric acid) or mono-unsaturated (oleic acid) FA as sole carbon sources. When tested on oleic acid, strains complemented with *SCOfadAB_2* and *SCOfadAB_3* exhibited slower growth rates and a lower final biomass concentration (measured as final OD_600_) than the strain complemented with *SCOfadAB_1*, while *E. coli* Δ*fadAB* transformed with an empty plasmid was unable to grow. Since the heterologous expression of the three clusters produced comparable protein levels (Figure 2A), it seems possible that differences in the catalytic efficiency of the enzymes or the substrate selectivity of the *S. coelicolor* FadAB protein complexes could explain the growth differences of the *E. coli* complemented strains. It has been widely reported that different FadAB isoforms have different substrate specificities. In *E. coli*, Campbell et al. reported the existence of YfcYX complex, an anaerobic paralog of the canonical FadAB (Campbell et al., 2003). Although YfcYX is mainly expressed under anaerobic conditions, phenotypical analyses of mutant strains suggest that the substrate specificities of the two complexes complement one another. These authors propose a model in which FadBA complex degrades long-chain FA and releases short- and medium-chain length intermediates that are substrates for the YfcYX complex (Campbell et al., 2003). More restricted substrate specificity was reported for *M. tuberculosis* FadB, which needs the action of a trans-acting enoyl-CoA isomerase in order to completely degrade cis-unsaturated FA (Srivastava et al., 2015). In the case of *S. coelicolor* FadAB complexes, low efficiency or narrow substrate specificity range of the complexes in the *E. coli* context might account for the lower growth rates exhibited by strains complemented with *SCOfadAB_2* and *SCOfadAB_3* when oleic acid is the sole carbon source.

Neighbor-joining phylogenetic tree shows the grouping of *SCOfadAB_2* and *SCOfadAB_3* together with *M. tuberculosis* FadB and *R. equi* FadB. On the other hand, *SCOfadAB_1* was located close to different FadB from Gram (−) enterobacteria (Figure 1C). This suggests separate evolutionary origins for those two groups of clusters and their maintenance could indicate their necessity. In this sense, the different expression patterns obtained from *SCOfadAB* promoters support this idea. Either in solid or in liquid media, expression from promoters *SCOfadAB_1* and *SCOfadAB_3* is enhanced by oleic acid at stationary and exponential phases of growth, respectively (Figure 3). It is noticeable that *SCOfadAB_3* led to slow growth on oleic acid when expressed in *E. coli* Δ*fadAB* but, however, its expression in *S. coelicolor* responds to this FA. On the other hand, expression from the *SCOfadAB_2* promoter was only detected in solid medium with glucose as sole carbon source. In a context of high gene redundancy, it seems reasonable that genes encoding the same function could be subject to different regulation patterns (Alam et al., 2010). For instance, 24 of the 35 *M. tuberculosis fadE* paralogs are expressed within macrophages and they are classified into four groups which exhibit different and opposite expression patterns (Schnappinger et al., 2003; Lam et al., 2008). This behavior was also reported for other non-actinomycete bacteria which exhibit redundancy in β-oxidation genes. In *B. subtilis*, phenotypic evidence from knockout mutants accounts for the existence of at least one *yusJ* (*fadE*) paralog which might possess a different expression pattern (Matsuoka et al., 2007). In *Acinetobacter* sp. strain ADP1, growth studies and analysis of revertants indicate that dicarboxylic acyl-CoA dehydrogenase DcaA possesses paralogs believed to act on different chain-length substrates and to express differentially according to the availability of structurally different FA (Parke et al., 2001). In *E. coli*, expression of *yfcY* (*fadI*) and *yfcX* (*fadJ*) genes occurs mainly in anaerobic conditions of growth while *fadA* and *fadB* genes reach maximal expression during aerobic conditions (Campbell et al., 2003). However, the authors observed that, although the two sets of genes exhibit opposite expression profiles, fine regulation mechanisms ensure basal expression of both of them during aerobic growth. In this context, we hypothesize that the three *SCOfadAB* clusters identified here could exhibit basal expression, with *SCOfadAB_1* and *SCOfadAB_3* upregulated in presence of exogenous FA and *SCOfadAB_2* upregulated in presence of glucose. This is in agreement with previous reports that glucose favors the assimilation of long-chain FA, partly by the induction of FadD activity (Banchio and Gramajo, 1997). However, it is worth noticing that up-regulation of reporter fusions may not necessarily involve overproduction of the corresponding proteins.

It seems reasonable that the inactivation of β-oxidation would prevent TAG mobilization. Therefore, if *SCOfadAB* clusters are actually involved in β-oxidation, their inactivation should lead to TAG over-accumulation. We tested this hypothesis by the prediction of mutations that could increase carbon flux to TAG biosynthesis. Optknock based on m_iMK1208 identified mutations in β-oxidation reactions that were not predicted by simulation based on the original model (Table 3). Based on our previous results in *Streptomyces* lipid metabolism (Banchio and Gramajo, 1997; Rodríguez and Gramajo, 1999; Rodríguez et al., 2001; Rodriguez et al., 2012; Arabolaza et al., 2008, 2010; Gago et al., 2011; Comba et al., 2013, 2014), we consider that the prediction based on m_iMK1208 is more consistent than the one based on iMK1208.

Single and double *SCOfadAB* knockout mutants were constructed and phenotypically evaluated. All of them were able to grow on lauric acid and oleic acid as sole carbon sources, indicating that none of the three clusters is essential for exogenous FA assimilation. However, we cannot exclude the involvement of β-oxidation genes different than the ones studied here in degradation of the tested FA. Notably, all *SCOfadAB* knockout strains exhibited increased TAG accumulation (Figure 4A), which could be explained by two hypotheses: (1) *SCOfadAB* knockouts possess increased TAG biosynthesis, or (2) *SCOfadAB* knockouts possess decreased TAG mobilization. In contrast to TAG quantifications, the quantifications of free FA in the wild type as well as in the *SCOfadAB* mutant strains were not reliable even reproducible from sample to sample. We constantly observed unusually high variations in free FA pools, after organic solvent extraction and TLC fractionation, which may lead to misapprehensions in the interpretations. Particularly, the TLC presented in Figure 4A shows a high content of FFA in *SCOfadAB_1*^−^ mutant strain, which was not exhibited by neither of the biological triplicates.

Assays of radiolabeled acetate incorporation indicated that TAG biosynthesis occurs in mutant strains to the same extent as in wild type M145 in both exponential and late-stationary phases of growth. In agreement with that, pulse and chase experiments clearly showed that TAG mobilization is reduced in the mutant strains (Figure 4C). In these experiments, cells were grown and labeled with ^14^C-acetate in minimal medium with glucose as sole carbon source, and then transferred to a fresh medium which promoted TAG mobilization (containing no carbon source and ammonium as nitrogen source; Alvarez et al., 2000). Although in these conditions all mutants exhibited a high compromise in TAG mobilization, the stronger phenotypes shown by some mutants suggest different biological roles for the three *SCOfadAB* clusters identified. Consistently, the double mutant *SCOfadAB_1*^−^*SCOfadAB_3*^−^ showed a TAG content 4.3-fold higher than M145 strain under the same culture conditions. A similar phenotype was observed in the oleaginous yeast *Yarrowia lipolytica* (Dulermo and Nicaud, 2011). In that case, TAG accumulation increased notably when β-oxidation was blocked by deleting *pox1-6* (acyl-CoA dehydrogenases) or *mfe1* (*fadB* homolog) in a strain over-expressing glycerol-3-phosphate dehydrogenase GDP1. These authors also demonstrated that β-oxidation regulates TAG accumulation in *Y. lipolytica*, not only by affecting mobilization but also by regulating the expression of genes involved in TAG biosynthesis. They observed that the diacylglycerol acyltransferases DGA1 and DGA2 are drastically up-regulated when β-oxidation is blocked in a GPD1 over-expressing strain. Indeed, the only deletion of *pox1-6* or *mfe1* strongly increases the expression of the lysophosphatidic acid acyltransferase SCT1 (Dulermo and Nicaud, 2011). These findings raise the question about the involvement of β-oxidation in the expression of TAG biosynthetic genes in *S. coelicolor*. Therefore, assessing their expression in the *SCOfadAB* KO generated in this work represents an interesting perspective toward a deep understanding of the relevance of β-oxidation in lipid accumulation by oleaginous microorganisms.

## Author contributions

SM, JP, CA, HG, and AA designed all the experiments. SM and JP constructed the plasmid backbones and performed all the experiments except those regarding chemostat cultures and flux balance analysis. SM performed chemostat cultures and flux balance analysis. SM and AA wrote the manuscript. All the authors read, corrected, and approved the final manuscript.

### Conflict of interest statement

The authors declare that the research was conducted in the absence of any commercial or financial relationships that could be construed as a potential conflict of interest.

## Supplementary material

Supplementary Figure 1 Analysis of lipid composition in *S. coelicolor* single *SCOfadAB* knockout mutants grown in minimal medium. **(A)** Total lipids extracted from 2.5 mg of lyophilized cells obtained from samples at 40 and 90 h of SMM cultures of the indicated strains were analyzed on silica gel TLC plates developed in hexane/diethylether/acetic acid (80:20:1, v/v/v), using Cu-phosphoric solution as visualization reagent. **(B)** Relative TAG content of the wild type and single mutant strains analyzed in **(A)**. Three independent total lipids TLC analysis were digitalized for each strain and the spots were quantified using ImageJ v1.48 software. The densitometry value obtained for TAG content of M145 strain at 40 h of growth was assigned the value of 1.

Supplementary Figure 2 Relative TAG and FFA content of the wild type and double mutant strains analyzed in the pulse-chase experiment shown in Figure 4C. Three independent total lipids TLC analysis were digitalized for each strain and the spots were quantified using ImageJ v1.48 software. The densitometry value obtained for TAG and FFA content of M145 strain at 40 h of growth was assigned the value of 1.

Supplementary Figure 3 Pulse-chase analysis of TAG from *S. coelicolor* wild type and single *SCOfadAB* knockout mutant strains after switching cultures to a SMM minimal medium without carbon source. Cell samples were collected immediately after medium shift (40 h) and subsequently, at the indicated time points. Total lipids were extracted from 2.5 mg of lyophilized [14C]-acetic acid-pulse labeled cells of each *S. coelicolor* strain and analyzed on silica gel TLC plates developed in hexane/diethylether/acetic acid (80:20:1, v/v/v). Radiolabeled lipid species were visualized using a PhosphoImager Screen.

Supplementary Figure 4 Fatty acid composition of TAG isolated from wild-type M145 **(A)** and *SCOfadAB_1*^−^ *SCOfadAB_3*^−^ double mutant strain **(B)**. Cells were cultivated to the stationary phase of growth (90 h) in R5 medium. Total lipid extracts from lyophilized mycelium were fractionated by preparative TLC and developed in hexane-diethylether-acetic acid (80:20:1, vol/vol/vol), and TAG was purified prior to subjection to gas chromatography analysis. Above each bar is indicated the relative abundance (percentage) of the corresponding fatty acid species.

## References

[B1] Alam M. T. Merlo M. E. Hodgson D. A. Wellington E. M. H. Takano E. Breitling R. (2010). Metabolic modeling and analysis of the metabolic switch in *Streptomyces coelicolor*. BMC Genomics 11:202 10.1186/1471-2164-11-202 PMC2853524 20338070

[B2] Alvarez H. Steinbüchel A. (2002). Triacylglycerols in prokaryotic microorganisms. Appl. Microbiol. Biotechnol. 60, 367–376. 10.1007/s00253-002-1135-0 12466875

[B3] Alvarez H. M. (2016). Triacylglycerol and wax ester-accumulating machinery in prokaryotes. Biochimie 120, 28–39. 10.1016/j.biochi.2015.08.016 26343555

[B4] Alvarez H. M. Kalscheuer R. Steinbüchel A. (2000). Accumulation and mobilization of storage lipids by *Rhodococcus opacus* PD630 and Rhodococcus ruber NCIMB 40126. Appl. Microbiol. Biotechnol. 54, 218–223. 10.1007/s002530000395 10968636

[B5] Arabolaza A. D'Angelo M. Comba S. Gramajo H. (2010). FasR, a novel class of transcriptional regulator, governs the activation of fatty acid biosynthesis genes in *Streptomyces coelicolor*. Mol. Microbiol. 78, 47–63. 10.1111/j.1365-2958.2010.07274.x 20624224

[B6] Arabolaza A. Rodriguez E. Altabe S. Alvarez H. Gramajo H. (2008). Multiple pathways for triacylglycerol biosynthesis in *Streptomyces coelicolor*. Appl. Environ. Microbiol. 74, 2573–2582. 10.1128/AEM.02638-07 18310412 PMC2394905

[B7] Banchio C. Gramajo H. C. (1997). Medium- and long-chain fatty acid uptake and utilization by *Streptomyces coelicolor* A3(2): first characterization of a gram-positive bacterial system. Microbiology 143(Pt 7), 2439–2447. 10.1099/00221287-143-7-2439 9245824

[B8] Bierman M. Logan R. O'Brien K. Seno E. T. Rao R. N. Schoner B. E. (1992). Plasmid cloning vectors for the conjugal transfer of DNA from *Escherichia coli* to *Streptomyces* spp. Gene 116, 43–49. 10.1016/0378-1119(92)90627-2 1628843

[B9] Bishop A. Fielding S. Dyson P. Herron P. (2004). Systematic insertional mutagenesis of a streptomycete genome: a link between osmoadaptation and antibiotic production. Genome Res. 14, 893–900. 10.1101/gr.1710304 15078860 PMC479117

[B10] Black P. N. DiRusso C. C. Metzger A. K. Heimert T. L. (1992). Cloning, sequencing, and expression of the fadD gene of *Escherichia coli* encoding acyl coenzyme A synthetase. J. Biol. Chem. 267, 25513–25520. 1460045

[B11] Bligh E. G. Dyer W. J. (1959). A rapid method of total lipid extraction and purification. Can. J. Biochem. Physiol. 37, 911–917. 10.1139/o59-099 13671378

[B12] Bradford M. M. (1976). A rapid and sensitive method for the quantitation of microgram quantities of protein utilizing the principle of protein-dye binding. Anal. Biochem. 72, 248–254. 10.1016/0003-2697(76)90527-3 942051

[B13] Burgard A. P. Pharkya P. Maranas C. D. (2003). Optknock: a bilevel programming framework for identifying gene knockout strategies for microbial strain optimization. Biotechnol. Bioeng. 84, 647–657. 10.1002/bit.10803 14595777

[B14] Buttner M. J. Chater K. F. Bibb M. J. (1990). Cloning, disruption, and transcriptional analysis of three RNA polymerase sigma factor genes of *Streptomyces coelicolor* A3(2). J. Bacteriol. 172, 3367–3378. 10.1128/jb.172.6.3367-3378.1990 2160942 PMC209148

[B15] Campbell J. W. Morgan-Kiss R. M. Cronan J. E. (2003). A new *Escherichia coli* metabolic competency: growth on fatty acids by a novel anaerobic beta-oxidation pathway. Mol. Microbiol. 47, 793–805. 10.1046/j.1365-2958.2003.03341.x 12535077

[B16] Cherepanov P. P. Wackernagel W. (1995). Gene disruption in *Escherichia coli*: TcR and KmR cassettes with the option of Flp-catalyzed excision of the antibiotic-resistance determinant. Gene 158, 9–14. 10.1016/0378-1119(95)00193-A 7789817

[B17] Comba S. Menendez-Bravo S. Arabolaza A. Gramajo H. (2013). Identification and physiological characterization of phosphatidic acid phosphatase enzymes involved in triacylglycerol biosynthesis in *Streptomyces coelicolor*. Microb. Cell Fact. 12:9 10.1186/1475-2859-12-9 PMC3599759 23356794

[B18] Comba S. Sabatini M. Menendez-Bravo S. Arabolaza A. Gramajo H. (2014). Engineering a *Streptomyces coelicolor* biosynthesis pathway into *Escherichia coli* for high yield triglyceride production. Biotechnol. Biofuels 7, 1–11. 10.1186/s13068-014-0172-0 25593590 PMC4295399

[B19] Datsenko K. A. Wanner B. L. (2000). One-step inactivation of chromosomal genes in *Escherichia coli* K-12 using PCR products. Proc. Natl. Acad. Sci. U.S.A. 97, 6640–6645. 10.1073/pnas.120163297 10829079 PMC18686

[B20] DiRusso C. C. (1990). Primary sequence of the *Escherichia coli* fadBA operon, encoding the fatty acid-oxidizing multienzyme complex, indicates a high degree of homology to eucaryotic enzymes. J. Bacteriol. 172, 6459–6468. 10.1128/jb.172.11.6459-6468.1990 1699931 PMC526834

[B21] Dulermo T. Nicaud J.-M. (2011). Involvement of the G3P shuttle and β-oxidation pathway in the control of TAG synthesis and lipid accumulation in *Yarrowia lipolytica*. Metab. Eng. 13, 482–491. 10.1016/j.ymben.2011.05.002 21620992

[B23] Fernández-Martínez L. T. Del Sol R. Evans M. C. Fielding S. Herron P. R. Chandra G.. (2011). A transposon insertion single-gene knockout library and new ordered cosmid library for the model organism *Streptomyces coelicolor* A3(2). Anton. Van Leeuwenhoek 99, 515–522. 10.1007/s10482-010-9518-1 20945092

[B22] Folch J. Lees M. Sloane Stanley G. H. (1957). A simple method for the isolation and purification of total lipides from animal tissues. J. Biol. Chem. 226, 497–509. 13428781

[B24] Gago G. Diacovich L. Arabolaza A. Tsai S.-C. Gramajo H. (2011). Fatty acid biosynthesis in actinomycetes. FEMS Microbiol. Rev. 35, 475–497. 10.1111/j.1574-6976.2010.00259.x 21204864 PMC3079561

[B25] Ghisla S. Thorpe C. (2004). Acyl-CoA dehydrogenases. A mechanistic overview. Eur. J. Biochem. 271, 494–508. 10.1046/j.1432-1033.2003.03946.x 14728676

[B26] González-Cerón G. Licona P. Servín-González L. (2001). Modified xylE and xylTE reporter genes for use in Streptomyces: analysis of the effect of xylT. FEMS Microbiol. Lett. 196, 229–234. 10.1016/S0378-1097(01)00073-8 11267784

[B27] Gouy M. Guindon S. Gascuel O. (2010). SeaView version 4: a multiplatform graphical user interface for sequence alignment and phylogenetic tree building. Mol. Biol. Evol. 27, 221–224. 10.1093/molbev/msp259 19854763

[B28] Hanahan D. (1983). Studies on transformation of *Escherichia coli* with plasmids. J. Mol. Biol. 166, 557–580. 10.1016/S0022-2836(83)80284-8 6345791

[B29] Herron P. R. Hughes G. Chandra G. Fielding S. Dyson P. J. (2004). Transposon Express, a software application to report the identity of insertions obtained by comprehensive transposon mutagenesis of sequenced genomes: analysis of the preference for *in vitro* Tn5 transposition into GC-rich DNA. Nucleic Acids Res. 32, e113. 10.1093/nar/gnh112 15308758 PMC514396

[B30] Hobbs D. H. Kim H. J. Chater K. F. Hills M. J. (1997). Mutants of lipid synthesis in *Streptomyces coelicolor*. Biochem. Soc. Trans. 25:S674. 10.1042/bst025s674 9450102

[B31] Jeong Y. Kim J.-N. Kim M. W. Bucca G. Cho S. Yoon Y. J.. (2016). The dynamic transcriptional and translational landscape of the model antibiotic producer *Streptomyces coelicolor* A3(2). Nat. Commun. 7:11605. 10.1038/ncomms11605 27251447 PMC4895711

[B32] Kelly B. G. Wall D. M. Boland C. A. Meijer W. G. (2002). Isocitrate lyase of the facultative intracellular pathogen *Rhodococcus equi*. Microbiology 148, 793–798. 10.1099/00221287-148-3-793 11882714

[B33] Khairon R. Zin N. M. Abdul Rahman M. Basri D. F. (2016). Comparative proteomic analysis of differential proteins in response to aqueous extract of quercus infectoria gall in methicillin-resistant *Staphylococcus aureus*. Int. J. Proteomics 2016:4029172 10.1155/2016/4029172 PMC5027293 27688912

[B34] Kieser T. Bibb M. J. Buttner M. J. Chater K. F. Hopwood D. A. (2000). Practical Streptomyces Genetics. Norwich: John Innes Foundation.

[B35] Kim J.-J. P. Battaile K. P. (2002). Burning fat: the structural basis of fatty acid beta-oxidation. Curr. Opin. Struct. Biol. 12, 721–728. 10.1016/S0959-440X(02)00390-1 12504675

[B36] Kim M. Sang Yi J. Kim J. Kim J.-N. Kim M. W. Kim B.-G. (2014). Reconstruction of a high-quality metabolic model enables the identification of gene overexpression targets for enhanced antibiotic production in *Streptomyces coelicolor* A3(2). Biotechnol. J. 9, 1185–1194. 10.1002/biot.201300539 24623710

[B37] Lam T. H. J. Yuen K. Y. Ho P. L. Wong K. C. Leong W. M. Law H. K. W.. (2008). Differential fadE28 expression associated with phenotypic virulence of *Mycobacterium tuberculosis*. Microb. Pathog. 45, 12–17. 10.1016/j.micpath.2008.01.006 18486437

[B38] Ledesma-Amaro R. Nicaud J.-M. (2016). *Yarrowia lipolytica* as a biotechnological chassis to produce usual and unusual fatty acids. Prog. Lipid Res. 61, 40–50. 10.1016/j.plipres.2015.12.001 26703186

[B39] Lennen R. M. Pfleger B. F. (2013). Microbial production of fatty acid-derived fuels and chemicals. Curr. Opin. Biotechnol. 24, 1044–1053. 10.1016/j.copbio.2013.02.028 23541503 PMC3834110

[B40] Lynch M. D. (2016). Into new territory: improved microbial synthesis through engineering of the essential metabolic network. Curr. Opin. Biotechnol. 38, 106–111. 10.1016/j.copbio.2016.01.009 26874263

[B41] Lyutzkanova D. Distler J. Altenbuchner J. (1997). A spectinomycin resistance determinant from the spectinomycin producer *Streptomyces flavopersicus*. Microbiology 143(Pt 7), 2135–2143. 10.1099/00221287-143-7-2135 9245803

[B42] Marcos A. T. Gutiérrez S. Díez B. Fernández F. J. Oguiza J. A. Martín J. F. (1995). Three genes hrdB, hrdD and hrdT of *Streptomyces griseus* IMRU 3570, encoding sigma factor-like proteins, are differentially expressed under specific nutritional conditions. Gene 153, 41–48. 10.1016/0378-1119(94)00759-L 7883183

[B43] Matsuoka H. Hirooka K. Fujita Y. (2007). Organization and function of the YsiA regulon of *Bacillus subtilis* involved in fatty acid degradation. J. Biol. Chem. 282, 5180–5194. 10.1074/jbc.M606831200 17189250

[B44] Olukoshi E. R. Packter N. M. (1994). Importance of stored triacylglycerols in Streptomyces: possible carbon source for antibiotics. Microbiology 140, 931–943. 10.1099/00221287-140-4-931 8012610

[B45] Parke D. Garcia M. A. Ornston L. N. (2001). Cloning and genetic characterization of dca genes required for beta-oxidation of straight-chain dicarboxylic acids in *Acinetobacter* sp. strain ADP1. Appl. Environ. Microbiol. 67, 4817–4827. 10.1128/AEM.67.10.4817-4827.2001 11571189 PMC93236

[B46] Peralta-Yahya P. P. Zhang F. del Cardayre S. B. Keasling J. D. (2012). Microbial engineering for the production of advanced biofuels. Nature 488, 320–328. 10.1038/nature11478 22895337

[B47] Pramanik A. Pawar S. Antonian E. Schulz H. (1979). Five different enzymatic activities are associated with the multienzyme complex of fatty acid oxidation from *Escherichia coli*. J. Bacteriol. 137, 469–473. 368024 10.1128/jb.137.1.469-473.1979 PMC218472

[B48] Raman N. Black P. N. DiRusso C. C. (1997). Characterization of the fatty acid-responsive transcription factor FadR. Biochemical and genetic analyses of the native conformation and functional domains. J. Biol. Chem. 272, 30645–30650. 10.1074/jbc.272.49.30645 9388199

[B49] Rodríguez E. Banchio C. Diacovich L. Bibb M. J. Gramajo H. (2001). Role of an essential acyl coenzyme A carboxylase in the primary and secondary metabolism of *Streptomyces coelicolor* A3(2). Appl. Environ. Microbiol. 67, 4166–4176. 10.1128/AEM.67.9.4166-4176.2001 11526020 PMC93144

[B50] Rodríguez E. Gramajo H. (1999). Genetic and biochemical characterization of the alpha and beta components of a propionyl-CoA carboxylase complex of *Streptomyces coelicolor* A3(2). Microbiology 145(Pt 1), 3109–3119. 10.1099/00221287-145-11-3109 10589718

[B51] Rodriguez E. Navone L. Casati P. Gramajo H. (2012). Impact of malic enzymes on antibiotic and triacylglycerol production in *Streptomyces coelicolor*. Appl. Environ. Microbiol. 78, 4571–4579. 10.1128/AEM.00838-12 22544242 PMC3370476

[B52] Röttig A. Hauschild P. Madkour M. H. Al-Ansari A. M. Almakishah N. H. Steinbüchel A. (2016a). Analysis and optimization of triacylglycerol synthesis in novel oleaginous Rhodococcus and Streptomyces strains isolated from desert soil. J. Biotechnol. 225, 48–56. 10.1016/j.jbiotec.2016.03.040 27034020

[B53] Röttig A. Strittmatter C. S. Schauer J. Hiessl S. Poehlein A. Daniel R.. (2016b). Role of wax ester synthase/acyl coenzyme a:diacylglycerol acyltransferase in oleaginous *Streptomyces* sp. strain G25. Appl. Environ. Microbiol. 82, 5969–5981. 10.1128/AEM.01719-16 27474711 PMC5038041

[B54] Sambrok J. Fritsch F. (1989). Maniatis T: Molecular Cloning: A Laboratory Manual. Cold Spring Harbor, NY: Cold Spring Harbor Laboratory Press.

[B55] Schellenberger J. Que R. Fleming R. M. T. Thiele I. Orth J. D. Feist A. M.. (2011). Quantitative prediction of cellular metabolism with constraint-based models: the COBRA Toolbox v2.0. Nat. Protoc. 6, 1290–1307. 10.1038/nprot.2011.308 21886097 PMC3319681

[B56] Schnappinger D. Ehrt S. Voskuil M. I. Liu Y. Mangan J. A. Monahan I. M.. (2003). Transcriptional adaptation of *Mycobacterium tuberculosis* within macrophages: insights into the phagosomal environment. J. Exp. Med. 198, 693–704. 10.1084/jem.20030846 12953091 PMC2194186

[B57] Schulz H. (1991). Beta oxidation of fatty acids. Biochim. Biophys. Acta 1081, 109–120. 10.1016/0005-2760(91)90015-A 1998729

[B58] Shiina T. Tanaka K. Takahashi H. (1991). Sequence of hrdB, an essential gene encoding sigma-like transcription factor of *Streptomyces coelicolor* A3(2): homology to principal sigma factors. Gene 107, 145–148. 10.1016/0378-1119(91)90308-X 1840545

[B59] Srivastava S. Chaudhary S. Thukral L. Shi C. Gupta R. D. Gupta R.. (2015). Unsaturated lipid assimilation by mycobacteria requires auxiliary cis-trans enoyl CoA isomerase. Chem. Biol. 22, 1577–1587. 10.1016/j.chembiol.2015.10.009 26628360

[B60] Volodina E. Steinbüchel A. (2014). (S)-3-hydroxyacyl-CoA dehydrogenase/enoyl-CoA hydratase (FadB') from fatty acid degradation operon of *Ralstonia eutropha* H16. AMB Express 4:69. 10.1186/s13568-014-0069-0 25401070 PMC4230905

[B61] Wältermann M. Luftmann H. Baumeister D. Kalscheuer R. Steinbüchel A. (2000). *Rhodococcus opacus* strain PD630 as a new source of high-value single-cell oil? Isolation and characterization of triacylglycerols and other storage lipids. Microbiology 146, 1143–1149. 10.1099/00221287-146-5-1143 10832641

[B62] Yagüe P. Rodríguez-García A. López-García M. T. Martín J. F. Rioseras B. Sánchez J.. (2013). Transcriptomic analysis of *Streptomyces coelicolor* differentiation in solid sporulating cultures: first compartmentalized and second multinucleated mycelia have different and distinctive transcriptomes. PLoS ONE 8:e60665. 10.1371/journal.pone.0060665 23555999 PMC3610822

[B63] Yagüe P. Rodríguez-García A. López-García M. T. Rioseras B. Martín J. F. Sánchez J.. (2014). Transcriptomic analysis of liquid non-sporulating *Streptomyces coelicolor* cultures demonstrates the existence of a complex differentiation comparable to that occurring in solid sporulating cultures. PLoS ONE 9:e86296. 10.1371/journal.pone.0086296 24466012 PMC3897704

[B64] Yang S. Y. He X. Y. (1999). Molecular mechanisms of fatty acid beta-oxidation enzyme catalysis. Adv. Exp. Med. Biol. 466, 133–143. 10.1007/0-306-46818-2_15 10709637

[B65] Yang S. Y. Li J. M. He X. Y. Cosloy S. D. Schulz H. (1988). Evidence that the fadB gene of the fadAB operon of *Escherichia coli* encodes 3-hydroxyacyl-coenzyme A (CoA) epimerase, delta 3-cis-delta 2-trans-enoyl-CoA isomerase, and enoyl-CoA hydratase in addition to 3-hydroxyacyl-CoA dehydrogenase. J. Bacteriol. 170, 2543–2548. 10.1128/jb.170.6.2543-2548.1988 3286611 PMC211169

[B66] Yang S. Y. Yang X. Y. Healy-Louie G. Schulz H. Elzinga M. (1990). Nucleotide sequence of the fadA gene. Primary structure of 3-ketoacyl-coenzyme A thiolase from *Escherichia coli* and the structural organization of the fadAB operon. J. Biol. Chem. 265, 10424–10429. 2191949

