# Supplementary figures and images for: Identification of FadAB Complexes Involved in Fatty Acid β-Oxidation in Streptomyces coelicolor and Construction of a Triacylglycerol Overproducing strain
Source: Front Microbiol. 2017 Aug 2;8:1428. doi: 10.3389/fmicb.2017.01428 (PMC5539140; doi:10.3389/fmicb.2017.01428)

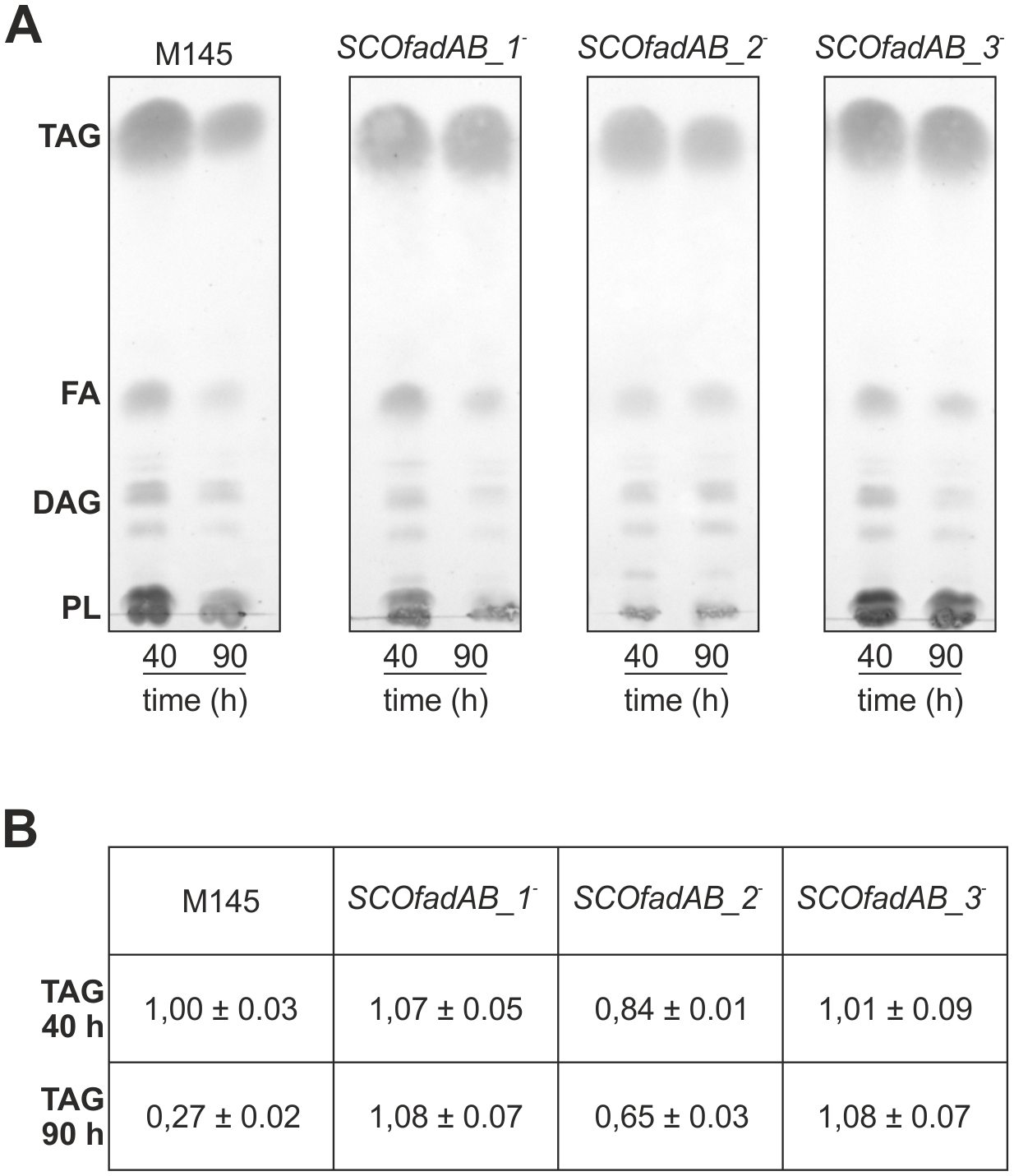

Supplement: Supplementary Figure 1 — Analysis of lipid composition in S. coelicolor single SCOfadAB knockout mutants grown in minimal medium. (A) Total lipids extracted from 2.5 mg of lyophilized cells obtained from samples at 40 and 90 h of SMM cultures of the indicated strains were analyzed on silica gel TLC plates developed in hexane/diethylether/acetic acid (80:20:1, v/v/v), using Cu-phosphoric solution as visualization reagent. (B) Relative TAG content of the wild type and single mutant strains analyzed in (A). Three independent total lipids TLC analysis were digitalized for each strain and the spots were quantified using ImageJ v1.48 software. The densitometry value obtained for TAG content of M145 strain at 40 h of growth was assigned the value of 1. [file Image1.TIF]

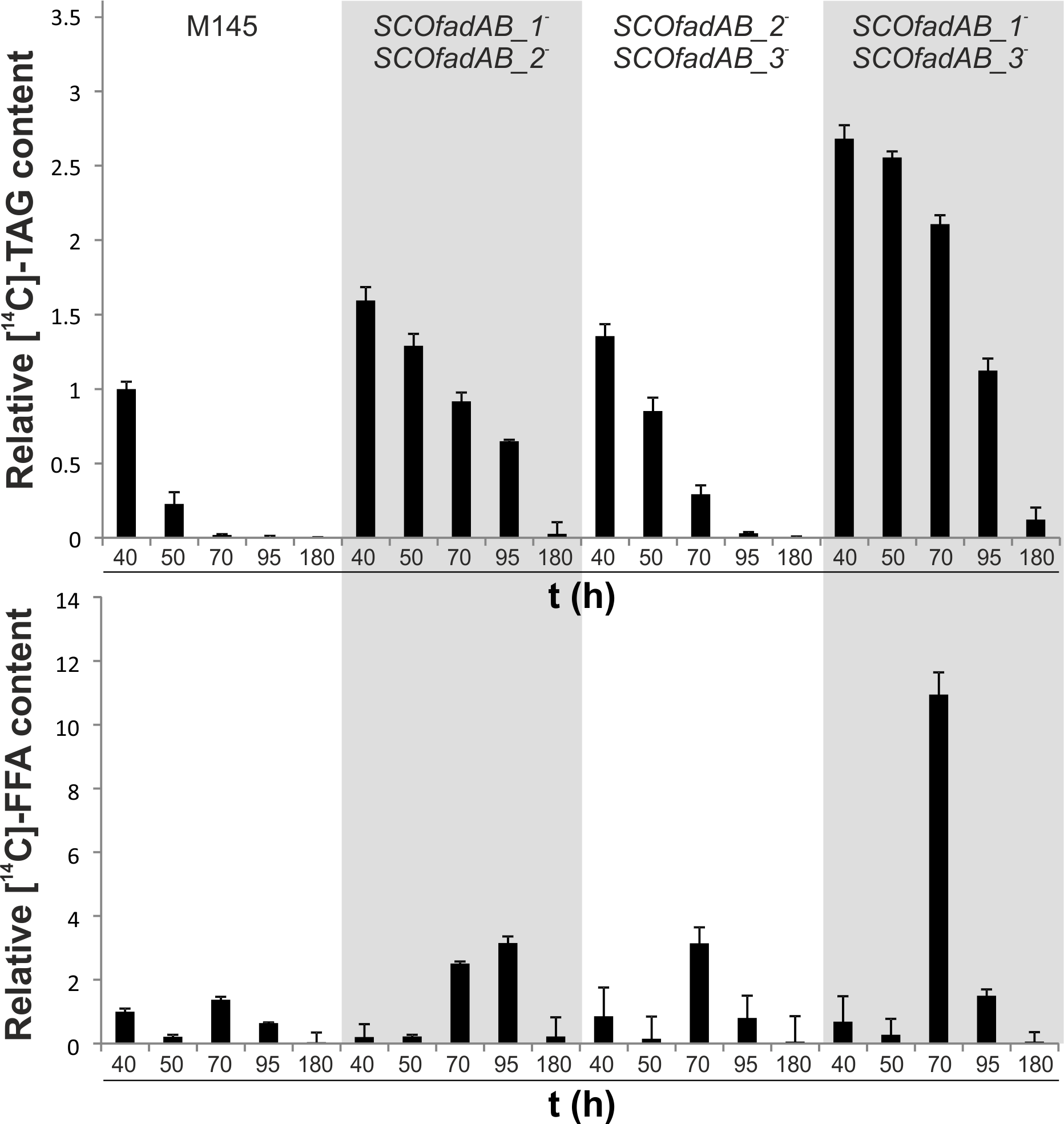

Supplement: Supplementary Figure 2 — Relative TAG and FFA content of the wild type and double mutant strains analyzed in the pulse-chase experiment shown in Figure 4C. Three independent total lipids TLC analysis were digitalized for each strain and the spots were quantified using ImageJ v1.48 software. The densitometry value obtained for TAG and FFA content of M145 strain at 40 h of growth was assigned the value of 1. [file Image2.TIF]

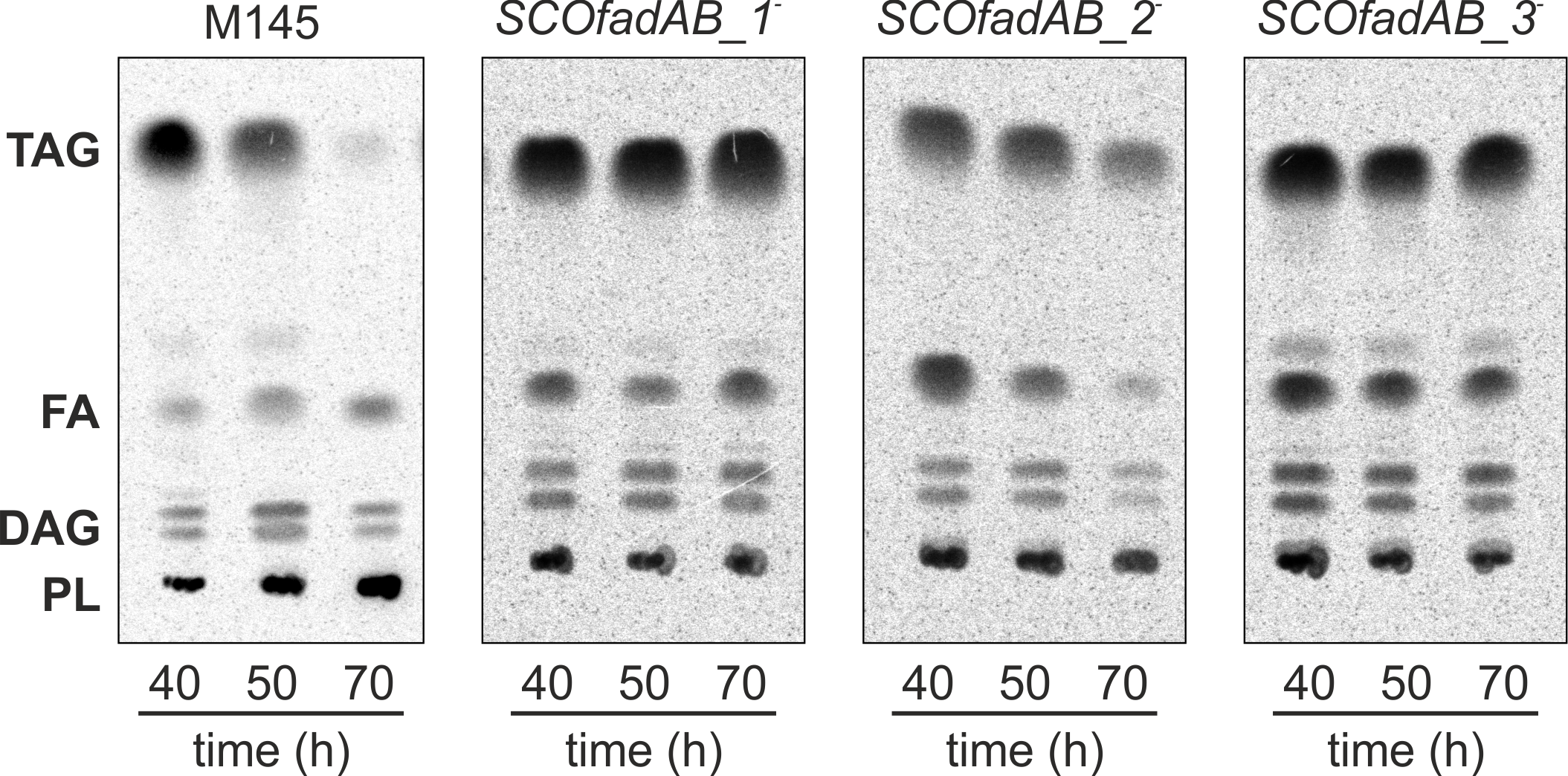

Supplement: Supplementary Figure 3 — Pulse-chase analysis of TAG from S. coelicolor wild type and single SCOfadAB knockout mutant strains after switching cultures to a SMM minimal medium without carbon source. Cell samples were collected immediately after medium shift (40 h) and subsequently, at the indicated time points. Total lipids were extracted from 2.5 mg of lyophilized [14C]-acetic acid-pulse labeled cells of each S. coelicolor strain and analyzed on silica gel TLC plates developed in hexane/diethylether/acetic acid (80:20:1, v/v/v). Radiolabeled lipid species were visualized using a PhosphoImager Screen. [file Image3.TIF]

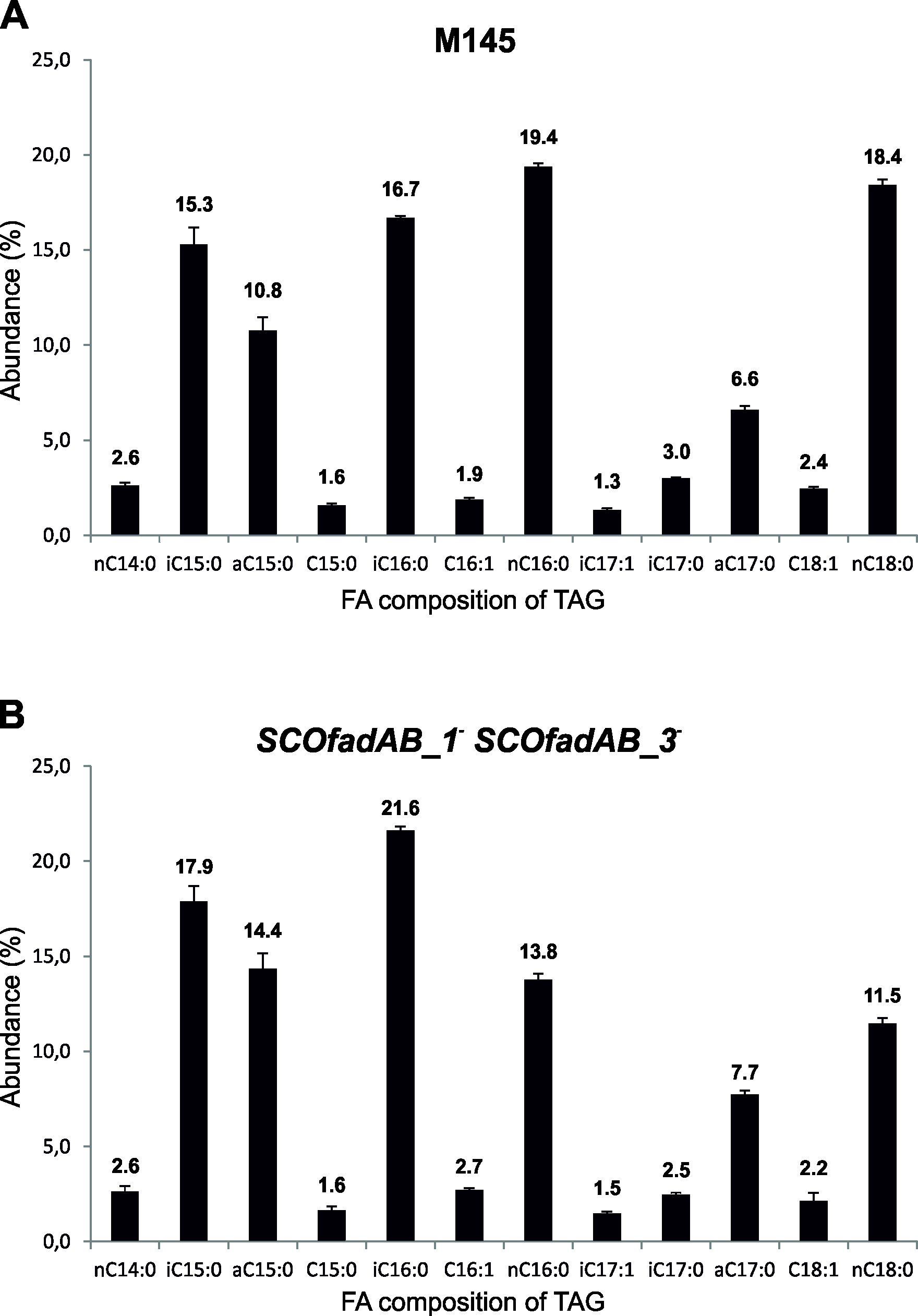

Supplement: Supplementary Figure 4 — Fatty acid composition of TAG isolated from wild-type M145 (A) and SCOfadAB_1− SCOfadAB_3− double mutant strain (B). Cells were cultivated to the stationary phase of growth (90 h) in R5 medium. Total lipid extracts from lyophilized mycelium were fractionated by preparative TLC and developed in hexane-diethylether-acetic acid (80:20:1, vol/vol/vol), and TAG was purified prior to subjection to gas chromatography analysis. Above each bar is indicated the relative abundance (percentage) of the corresponding fatty acid species. [file Image4.TIF]
